# Silencing of six susceptibility genes results in potato late blight resistance

**DOI:** 10.1007/s11248-016-9964-2

**Published:** 2016-05-28

**Authors:** Kaile Sun, Anne-Marie A. Wolters, Jack H. Vossen, Maarten E. Rouwet, Annelies E. H. M. Loonen, Evert Jacobsen, Richard G. F. Visser, Yuling Bai

**Affiliations:** Wageningen UR Plant Breeding, Wageningen University and Research Centre, Droevendaalsesteeg 1, 6708 PB Wageningen, The Netherlands

**Keywords:** Late blight, Potato, Resistance, RNAi, Susceptibility gene

## Abstract

*Phytophthora infestans*, the causal agent of late blight, is a major threat to commercial potato production worldwide. Significant costs are required for crop protection to secure yield. Many dominant genes for resistance (*R*-genes) to potato late blight have been identified, and some of these *R*-genes have been applied in potato breeding. However, the *P. infestans* population rapidly accumulates new virulent strains that render *R*-genes ineffective. Here we introduce a new class of resistance which is based on the loss-of-function of a susceptibility gene (*S*-gene) encoding a product exploited by pathogens during infection and colonization. Impaired *S*-genes primarily result in recessive resistance traits in contrast to recognition-based resistance that is governed by dominant *R*-genes. In *Arabidopsis thaliana*, many *S*-genes have been detected in screens of mutant populations. In the present study, we selected 11 *A. thalianaS*-genes and silenced orthologous genes in the potato cultivar Desiree, which is highly susceptible to late blight. The silencing of five genes resulted in complete resistance to the *P. infestans* isolate Pic99189, and the silencing of a sixth *S*-gene resulted in reduced susceptibility. The application of *S*-genes to potato breeding for resistance to late blight is further discussed.

**Electronic supplementary material:**

The online version of this article (doi:10.1007/s11248-016-9964-2) contains supplementary material, which is available to authorized users.

## Introduction

**Fig. 1:**
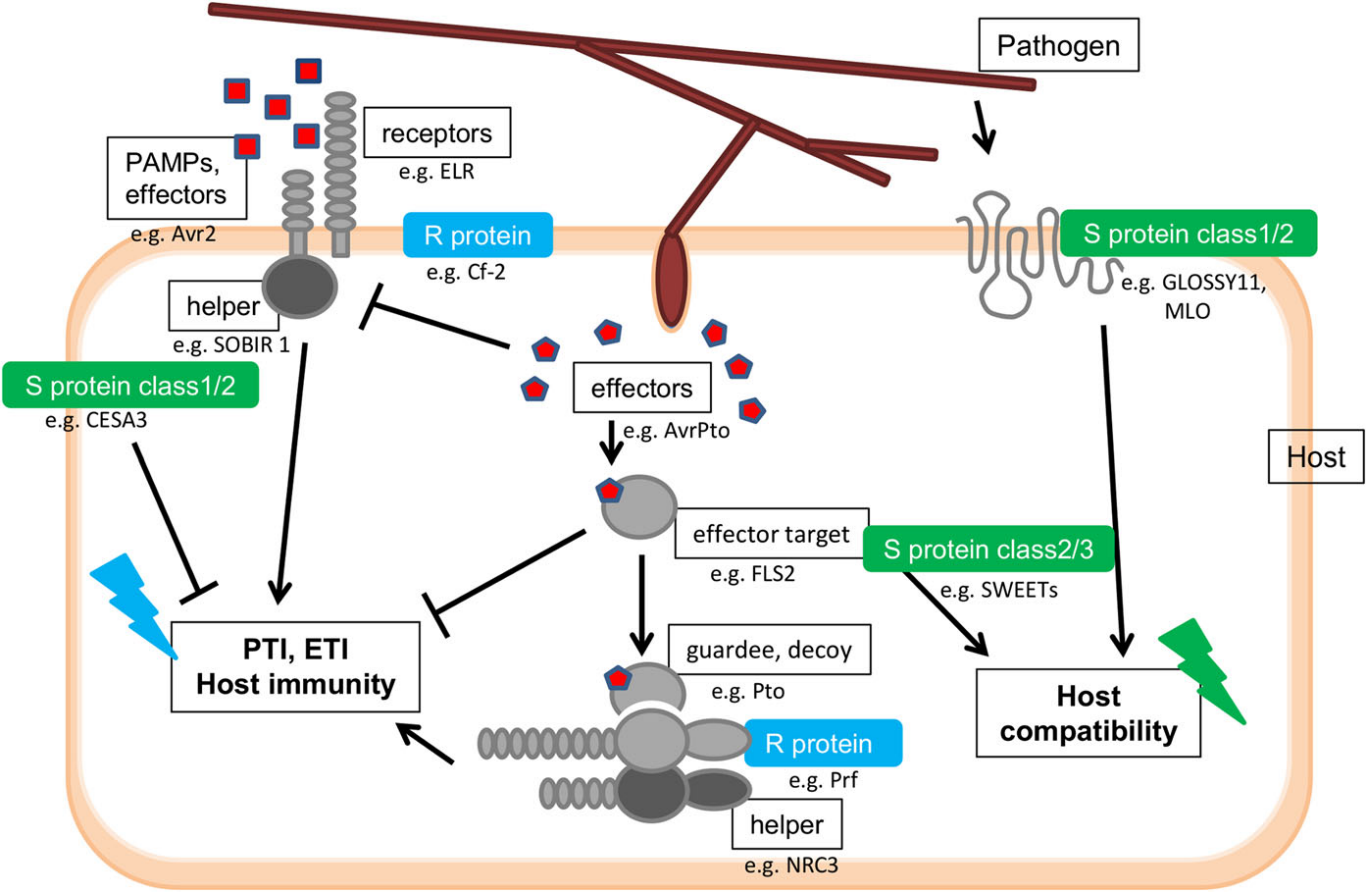
Plant innate immunity: PAMP-triggered immunity (PTI) and effector-triggered immunity (ETI). Apoplastic pathogen-associated molecular patterns (PAMPs/apoplastic effectors), intracellular effectors or modified effector targets are actively perceived by receptors in the plasma membrane or resistance (R) proteins in the cytoplasm, resulting in the activation of PAMP-triggered immunity (PTI) and effector-triggered immunity (ETI). Helper proteins and guard proteins/decoys are involved in the co-perception of pathogen-derived components (Césari et al. 2014). Pathogens use host proteins (S proteins) encoded by plant susceptibility genes (*S*-genes) to facilitate entry and growth, resulting in host compatibility (Doehlemann and Hemetsberger 2013). MLO, CESA3 and SWEETs are examples of S proteins in class 1, 2, or 3 according to van Schie and Takken (2014). Class 1 genes provide features that facilitate the entrance of a pathogen, class 2 genes increase innate immunity when the gene is disabled, and class 3 genes encode substrates essential for the pathogen

The plant immune system comprises an intricate network of active and passive mechanisms that successfully prevent the colonization of a host by a pathogen (Jones and Dangl 2006) (Fig. 1). In many cases, defence is actively triggered upon first contact between a plant and pathogen. Plasma membrane receptors perceive pathogen-associated molecular patterns (PAMPs) or apoplastic effectors (Fig. 1). This perception leads to intracellular signal transduction events, culminating in defence responses that, when effective, induce PAMP-triggered immunity (PTI). A known example is the receptor-like protein ELR (elicitin response). ELR was isolated from the wild potato species *Solanum microdontum* and can mediate the broad-spectrum recognition of elicitins (referred to as oomycete PAMPs) from several *Phytophthora* species (Du et al. 2015). The second layer of defence relies on proteins encoded by resistance genes (*R*-genes) that recognize intracellular avirulence (Avr) effectors. This recognition results in effector-triggered immunity (ETI).

Potato late blight, caused by *Phytophthora infestans*, is considered to be the most serious potato disease worldwide. An asexual cycle of sporangial proliferation is completed within 5 days (Fry 2008). Depending on environmental conditions, an unprotected potato field with a susceptible cultivar (cv.) can be devastated within 10 days after infection with *P. infestans* (Fry 2008). The control of potato late blight is dependent on fungicide sprays and the use of cultivars carrying dominant *R*-genes (Haverkort et al. 2009). All late blight *R*-genes identified thus far belong to the coiled-coil nucleotide binding leucine-rich repeat (CC-NB-LRR or NLR) class and reside inside the plant cell where these genes recognize *P. infestans* avirulence effectors (Avr) of the RxLR class.

Because the resistance conferred by *R*-genes is, in general, race-specific, ETI can be broken due to the rapid evolution of pathogen effectors in agricultural practice (Vleeshouwers et al. 2011). For example, *Rpi*-*vnt1*, isolated from *S. venturii*, confers resistance to a broad spectrum of *P. infestans* lineages and races in European potato growing areas (Pel et al. 2009). However, this resistance can be overcome by the EC1 lineage, which is abundant in Ecuador. Thus, in addition to exploiting dominant *R*-genes, combinations of resistance traits that are effective against the prevailing *P. infestans* population are needed for durable late blight resistance. Therefore, pyramiding ETI receptors is expected to enhance resistance durability (Kim and Hwang 2012; Rietman et al. 2012; Zhu et al. 2013).

The resistance conferred by *R*-genes is based on pathogen recognition followed by the induction of defence responses. Another type of resistance, based on the loss-of-function of a susceptibility gene (*S*-gene), has recently been introduced (Eckardt 2002; Pavan et al. 2011). Plant genes are termed *S*-genes when a pathogen takes advantage of them for its own benefit during the colonization of the plant. Pathogens are impeded from colonizing the plant when these genes become dysfunctional as a result of a recessive mutation or non-expression. Thus, when disabled, *S*-genes can induce a resistance phenotype resembling that of healthy plants. For example, the loss-of-function mutant in the *Powdery Mildew Resistance 6* (*PMR6*) gene prevents powdery mildew growth (Vogel et al. 2002). Based on the mechanism, *S*-genes have been divided into three classes (van Schie and Takken 2014). The first class comprises the genes required in early pathogen infection steps. One example is the maize (*Zea mays*) wax mutant *glossy 11*, which limits powdery mildew (*Blumeria graminis*) spore germination (Hansjakob et al. 2011, Fig. 1). The second class of *S*-genes encodes negative regulators of plant immunity, such as the *CesA3* gene, which is involved in cellulose synthesis (Ellis et al. 2002; Ellis and Turner 2001, Fig. 1). The presence of homozygous recessive mutant alleles of the CesA3 gene can confer resistance to multiple pathogens, as a result of an increase of *in planta* levels of abscisic and jasmonic acid and ethylene. The third class of *S*-genes includes genes involved in pathogen sustenance, such as metabolite biosynthesis and sugar transport. For example, the *Downy Mildew Resistant 1* (*DMR1*) gene encodes homoserine kinase (HSK). Arabidopsis *dmr1* mutants are resistant to the downy mildew fungus *Hyaloperonospora parasitica* (Huibers et al. 2013; Van Damme et al. 2008; van Damme et al. 2009) and the fungi *Fusarium graminearum* and *F. culmorum*, which cause Fusarium Ear Blight (FEB) disease on small grain cereals (Brewer et al. 2014). Other examples include the genes encoding SWEET proteins, identified as factors that are required for susceptibility to *Xanthomonas oryzae*, which provide a carbon source to the pathogen (Chen et al. 2010; Streubel et al. 2013; Yuan et al. 2010, Fig. 1).

The aim of the present study was to impair *S*-genes in plants to obtain durable resistance (Pavan et al. 2010). In tomato, we showed that silencing *DMR1* and *PMR4* orthologues resulted in resistance to the powdery mildew fungus *Oidiumneolycopersici* (Huibers et al. 2013), suggesting that *S*-genes, such as *PMR4* and *DMR1*, are conserved among plant species and that impaired orthologues confer resistance to the associated pathogens in other plant species. This finding prompted us to identify potato orthologues of *S*-genes reported in other plant species (particularly *A. thaliana*) and to generate a proof of the *S*-gene concept in potato. We silenced 11 *S*-genes in potato via RNAi and showed that the silencing of five of these genes conferred complete resistance to late blight in potato, while the silencing of a sixth gene only conferred reduced susceptibility to this disease.

## Materials and methods

### Identification of potato orthologues of *S*-genes

Previously, tomato orthologues of 11 *A. thalianaS*-genes were identified as described in Huibers et al. (2013). Briefly, *A. thaliana* protein sequences were used as a query in a TBLASTN programme against the SGN Tomato Combined database (http://solgenomics.net/tools/blast/) to search for homologous sequences. The tomato and Arabidopsis amino acid sequences were aligned, and the tomato sequences that showed a high level of homology with the *A. thalianaS*-genes were considered orthologues. To identify potato orthologues, *A. thaliana* protein sequences were used in a BLASTP analysis at the Spud DB Potato Genomics Resource website (http://solanaceae.plantbiology.msu.edu/blast.shtml). Subsequently, protein and mRNA sequences with the lowest E-values were downloaded. Next, phylogenetic analyses were performed by aligning *A. thaliana*, tomato and potato protein sequences in MEGA5.1 (Tamura et al. 2011) using standard parameters (Supplementary Fig. 1). The tomato and potato genes showing the highest level of homology (on amino acid level) to *A. thaliana S*-genes were considered to be *S*-gene orthologues and were indicated with the prefix *Sl*- or *St*- to represent *S. lycopersicum* and *S. tuberosum*, respectively.

### Construction of the silencing vectors

The binary vector pHellsgate8 (CSIRO, Australia) has a kanamycin resistance gene as a selectable marker and was used to generate RNAi constructs (Helliwell and Waterhouse 2003; Waterhouse and Helliwell 2003). This vector contains a CaMV 35S promoter which drives the expression of the inverted repeat. For all genes, except *SR4*, *DND2*, and *PMR4*, primers were designed to amplify fragments of the target genes ranging from 150 to 300 bp from tomato cDNA sequences of the cv. Moneymaker (Supplementary Table 1). For *SR4* and *DND2*, primers were designed to amplify potato cDNA sequences of the cv. Desiree. The forward primer contained CACC at the 5′ end for directional cloning into the pENTR/D-TOPO vector (Thermo Fisher Scientific). Total RNA was isolated using a RNeasy kit from Qiagen (Germany). RNA was treated with RNAse-free DNase (Qiagen). Subsequently, this RNA was used as a template for cDNA synthesis using an iScript cDNA synthesis kit (Bio-Rad). The PCR products were amplified using Phusion High-Fidelity DNA polymerase (Thermo Fisher Scientific), inserted into the pENTR/D-TOPO cloning vector and transformed into One-Shot TOP10 *E. coli* cells. The plasmid DNA of the clones was sequenced to verify the insert. To generate the *SlPMR4* silencing construct (Huibers et al. 2013), we synthesized a 101-bp DNA fragment that was identical to the first 97 bp of the predicted coding sequence of Solyc07g053980 (the tomato *PMR4* ortholog, Supplementary Fig. 1) and contained CACC at the 5′ end flanked by attL sites in pUC57 (Genscript, USA). For *PMR6*, the silencing fragment was based on the coding sequence of tomato Solyc05g014000, but could potentially cross-silence multiple potato *PMR6*-like homologs (Sotub05g015080, Sotub11g012470, Sotub06g029220 and Sotub03g023350). The primer sequences are provided in Supplementary Table 1. For expression *in planta*, the RNAi fragments were transferred from the entry clone through an LR clonase reaction to the pHellsgate8 vector.

### Growth and development of transgenic potato plants

The tetraploid potato cv. Desiree (susceptible to late blight) was used for transformation according to the protocol of Visser et al. (1991). The transformants were transferred from MS medium (Murashige and Skoog 1962) supplemented with vitamins, 30 g l^−1^ sucrose and 100 mg l^−1^ kanamycin to similar fresh MS medium without kanamycin. After 3 weeks of growth at 24 °C and a light intensity of 100 W/m^2^, the rooted transformants were transferred to plastic pots containing potting soil in a growth compartment at 21 and 19 °C during 16-h days and 8-h nights, respectively. For each RNAi construct, more than eight independent primary transformants were randomly selected and cultured in a greenhouse and subsequently tested for resistance. Three biological replicates were grown for each transformant.

### Pathogen inoculations and detached leaflet assay (DLA)

The *P. infestans* isolate Pic99189 (race 1.2.5.7.10.11) (Flier et al. 2002) was used in the present study. For each experiment, the isolate was grown on rye agar medium supplemented with 2 % sucrose for 10–15 days at 15 °C in closed Petri dishes to induce sporangia formation (Caten and Jinks 1968). To release zoospores from sporangia, ice-cold tap water was added to the Petri dishes, followed by incubation for 3 h at 4 °C. The zoospore concentration was assessed by bright field microscopy using a Fuchs-Rosenthal counting chamber and adjusted to 5 × 10^4^ spores/ml. The resistance of potato RNAi transformants to Pic99189 was examined using a 10-µl droplet inoculation in detached leaflet assays (DLA) (Vleeshouwers et al. 1999). The leaves were harvested from plants after 5–6 weeks of greenhouse growth. The fourth or fifth fully developed leaf (counted from the top) was used. The lesion diameters were measured from 3–6 days post-inoculation using an electronic calliper (Helios DIGI-MET^®^).

### RNA isolation and quantitative real-time (qRT)-PCR

The kanamycin-resistant transformants were confirmed by PCR using Fw-NPTII and Rv-NPTII primers (Supplementary Table 1). The PCR-positive transformants were transferred to the greenhouse. More than eight independent transformants were randomly selected per gene, and the silencing levels of the transformants were evaluated by qRT-PCR using gene-specific primers (Supplementary Table 1, -qPCR), producing products of approximately 200 bp. Plant total RNA was extracted using a MagMAX-96 total RNA Isolation kit (Ambion). The quantity of the isolated RNA was measured using a Nanodrop Spectrophotometer ND-1000 (Isogen), and the cDNA was produced using an iScript cDNA synthesis kit (Bio-Rad). qRT-PCR was performed in triplicate using a C1000TM Thermal Cycler PCR system (Bio-Rad) with iQ SYBR Green supermix (Bio-Rad). The potato *EF1a* (Sotub06g010680) transcript was used as an internal control to determine the relative transcript levels. The relative level of gene expression was calculated using the $2^{{ -\Delta \Delta {\text{C}}_{\text{t}} }}$ method (Livak and Schmittgen 2001; Nicot et al. 2005). For the qRT-PCR assay, three technical replicates were included for each experiment, and the expression of each gene was investigated in three biological replicates.

## Results

### Identification of potential potato *S*-gene orthologues

To identify potato orthologues of the 11 *S*-genes listed in Table 1, we used the amino acid sequences of *A. thaliana* in a BLAST analysis of the potato sequence database. Potato sequences with an amino acid identity higher than 75 % were selected and used in phylogenetic studies (Supplementary Fig. 1). Based on multiple sequence alignments, sequences showing the highest degree of homology with the *S*-gene in *A. thaliana* were considered to be potential orthologues in potato (Table 2, column 2). The closest homolog to *AtSR1* in the potato database was Sotub01g012330, and silencing fragments were designed for this gene. However, when a subsequent TBLASTN search was conducted using the NCBI database, the closest homolog of *AtSR1* in the potato RefSeq_RNA database was XM_006355276.1, which corresponds to Sotub04g020530. This gene on chromosome 4 was closer to *AtSR1* in the phylogenetic tree than to Sotub01g012330 (Supplementary Fig. 1). Because Sotub01g012330 was closer to *AtSR4* and *SlSR4* (Yang et al. 2012), we referred to Sotub01g012330 as *StSR4* in Table 2.

**Table 1:** Selected *S*-genes identified in Arabidopsis

Gene name	Mutant resistance to pathogens						Mutant notes	Class^g^	References
	*PS* ^a^	PM^b^	*PP* ^c^	*HP* ^d^	*BC* ^e^	*AB* ^f^			
*CESA3*	nd	+	nd	nd	nd	nd	High level of resistance to the herbicide, gametophytic lethal	2	Ellis et al. (2002), Ellis and Turner (2001)
*DMR1*	nd	+	nd	+	nd	nd	Chlorosis and reduced growth	3	Huibers et al. (2013), Van Damme et al. (2005), van Damme et al. (2009)
*DMR6*	–	+	nd	+	nd	nd		2	Van Damme et al. (2005, 2008)
*DND1*	+	nd	nd	+	+	+	Smaller plant, early senescence, moderate lesion mimic	2	Ahn (2007), Clough et al. (2000), Genger et al. (2008), Govrin and Levine (2000), Jurkowski et al. (2004), Su’udi et al. (2011)
*SR1*	+	+	nd	nd	+	nd	Sensitive to herbivore attack, forms chlorotic lesions on leaf lamina	2	Doherty et al. (2009), Du et al. (2009), Galon et al. (2008), Kim et al. (2013), Laluk et al. (2012), Nie et al. (2012), Qiu et al. (2012)
*PMR4*	nd	+	nd	+	+	–	Resistance to the green peach aphid *Myzus persicae*, enhanced susceptibility to the fungal pathogens *Pythium irregulare*	2	Nishimura et al. (2003)
*BIK1*	+	nd	nd	nd	–	–	Altered root growth	2	Veronese et al. (2006)
*CPR5*	+	nd	+	nd	nd	nd	Spontaneous development of necrotic lesions; affected trichome development	2	Bowling et al. (1997), Jing et al. (2007), Jing and Dijkwel (2008), Love et al. (2007)
*DND2*	+	nd	nd	+	+	+	Smaller plant, early senescence, moderate lesion mimic	2	Ahn (2007), Clough et al. (2000), Genger et al. (2008), Govrin and Levine (2000), Jurkowski et al. (2004), Su’udi et al. (2011)
*PMR5*	–	+	–	nd	nd	nd	Smaller plants than wt; altered leaf morphology: leaves are shorter, rounder and cupped slightly upward compared to wt; Altered cell wall composition;	3	Chandran et al. (2013), Vogel et al. (2004)
*PMR6*	–	+	–	nd	nd	nd	Smaller plants than wt; altered leaf morphology: leaves are shorter, rounder and cupped slightly upward compared to wt; altered cell wall composition containing more pectin than wt, altered hydrogen bonding structure of cellulose	3	Chandran et al. (2013), Vogel et al. (2002)

Resistances conferred by mutated alleles, and phenotypic side effects are indicated per gene

+, significantly reduced susceptibility; –, no reduced susceptibility; nd, not determined

^a^ *Pseudomonas syringae*

^b^PM, powdery mildews: *Erysiphe* *cichoracearum*; *Erysiohe orontii*; *Oidium lycopersicum*; *Blumeria graminis*; *Golovinomyces cichoracearum*; *Golovinomyves orontii*

^c^ *Peronospora parasitica;*

^d^ *Hyaloperonospora parasitica*

^e^ *Botrytis cinerea*

^f^ *Alternaria brassicicola*

^g^According to van Schie and Takken (2014)

**Table 2:** Selected potato orthologs of 11 *S*-genes and their effects in transformants obtained after RNAi silencing

Gene name	Potato *S* gene homologs	No. plants							
		Tested by	Reduced transcription		DLA by Pic99189		Dwarfing	Autonecrosis	Color loss
		qPCR	>60 % (+)	<60 % (−)	R	S			
*CESA3*	*StCESA3*(Sotub01g026250)	8	5	3	5	3	–	–	–
*DMR1*	*StDMR1* (Sotub04g008400)	12	5	7	5	7	4+/8–	–	5+/7–
*DMR6*	*StDMR6* (Sotub06g027890)	12	6	6	4	8	–	–	–
*DND1*	*StDND1* (Sotub02g034320)	16	12	4	12	4	7+/9–	13+/3–	7+/9–
*SR1*	*StSR4* (Sotub01g012330)^a^	25	11	14	5	20	–	–	–
*PMR4*	*StPMR4* (Sotub07g019600)	27	13	14	8	19	–	–	–
*BIK1*	*StB1K1* (Sotub04g010100)	8	5	3	0	8	–	–	–
*CPR5*	*StCPR5* (Sotub04g022770)	12	4	8	0	12	4+/8–	–	4+/8–
*DND2*	*StDND2*(Sotub10g007010)	8	4	4	0	8	–	–	–
*PMR5*	*StPMR5* (Sotub06g006190)	27	10	17	0	27	–	–	–
*PMR6*	*StPMR6* (Sotub11g012470)	12	7	5	0	12	–	–	–

*DLA* detached leaf assay, *R* resistant, *S* susceptible

^a^See explanation in text

### Silencing of six potato *S*-genes results in reduced susceptibility to *P. infestans*

To assess the significance of these potential *S*-genes for susceptibility to *P. infestans* in potato, RNAi constructs of all of the selected potato orthologues were generated and used to transform the potato cv. Desiree, which is susceptible to late blight, using *Agrobacterium tumefaciens*-mediated transformation. From each of the 11 *S*-genes, several independent transformants with highly reduced transcript levels (>60 %) of the targeted *S*-gene were selected (Table 2, column 3, Supplementary Fig. 2). These transformants were further indicated as well-silenced transformants, and those with no or a low reduction in the transcript level (<60 %) of the targeted *S*-gene were considered to be negative controls in the following experiments (Table 2, column 4, Supplementary Fig. 2).

**Fig. 2:**
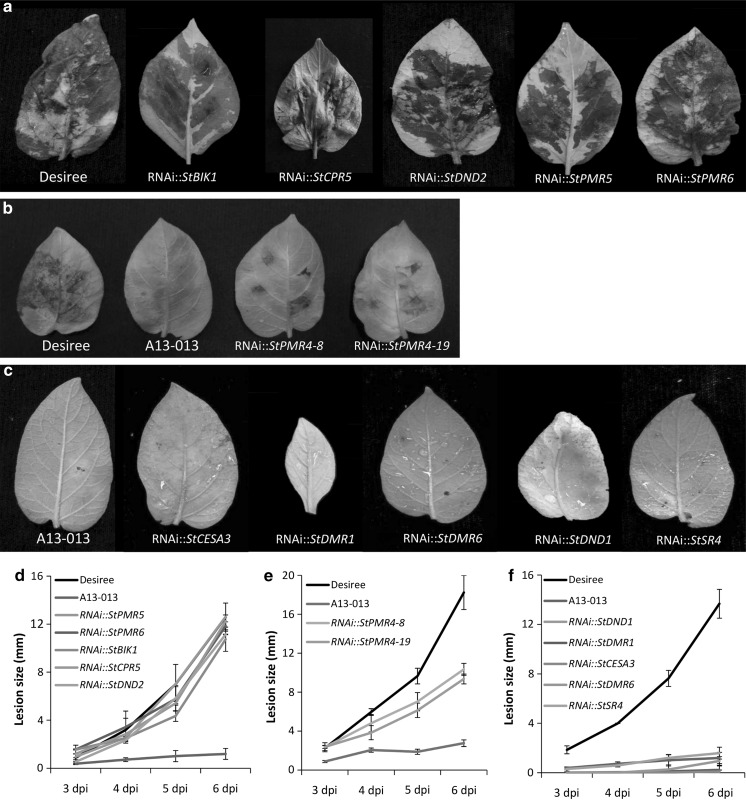
Detached leaf assay (DLA) of potato RNAi transformants with *Phytophthora infestans* isolate Pic99189. **a** Leaves of the five RNAi transformants (*StBIK1*, *StCPR5*, *StDND2*, *StPMR5*, *StPMR6*) showing a diseased phenotype similar to that of cv. Desiree at 7 days post inoculation (dpi). **b** Leaves of two independent RNAi::*StPMR4* transformants compared with the susceptible control Desiree and the resistant control A13-013 at 6 dpi. Both transformants #8 and #19 show strong silencing of *StPMR4* (Table 2). **c** Leaves of the resistant control A13-013 and the five RNAi transformants (*StCESA3*, *StDMR1*, *StDMR6*, *StDND1*, *StSR4*) showing no disease symptoms at 7 dpi. **d**–**f** Development of lesion size on the inoculated leaves in **a**, **b**, and **c**, respectively. Data were collected at 3, 4, 5 and 6 dpi. Untransformed Desiree plants were included as the susceptible control and A13-013 plants were included as the resistant control in this experiment. For each experiment, more than four well-silenced transformants per investigated gene were tested (three individual plants per transformant, one leaf per plant). Three independent experiments were performed with similar results

To determine whether the silencing of these genes impacted the susceptibility to late blight, the leaves of 4-to-5-week-old silenced plants were inoculated with the *P. infestans* isolate Pic99189. The leaves of cv. Desiree and A13-013 (a transformant containing the dominant resistance gene *Rpi*-*vnt1.1* in the genetic background of cv. Desiree and conferring resistance to Pic99189, Zhu et al. 2013) were used as susceptible and resistant controls, respectively. The inoculated leaves were visually inspected (Fig. 2a–c). 3–6 days post inoculation (dpi), the lesion size was measured, and lesion growth over time was plotted (Fig. 2d–f). Large lesions were observed on the inoculated leaves of the cv. Desiree and transformants in which *StBIK1*, *StCPR5*, *StDND2*, *StPMR5* or *StPMR6* were silenced (Fig. 2a). The lesion size showed a steady increase from 3 to 6 dpi (Fig. 2d).

Smaller lesions were observed on inoculated leaves of *StPMR4*-silenced potato plants compared with those of Desiree (Fig. 2b). At 6 dpi, the inoculated parts of Desiree leaves were completely blighted with obvious sporulation, while on the leaves of RNAi::*StPMR4* potato plants, the sporulation was only visible through a binocular. The lesion growth on the leaves of these transformants was much slower than on Desiree leaves (Fig. 2e). Thus, the silencing of *StPMR4* resulted in reduced susceptibility to *P. infestans*.

In contrast, up to 6 dpi, no lesion growth was visible on the leaves of resistant A13-013 control plants and transformants in which the five individual *S*-genes (*StCESA3*, *StDMR1*, *StDMR6*, *StDND1* and *StSR4*) were silenced (Fig. 2c, f). For each of these five genes, at least two independent well-silenced transformants (with three plants per transformation event) were tested (Table 2). All inoculated leaves showed similar results.

### Fitness costs associated with *S*-gene silencing

**Fig. 3:**
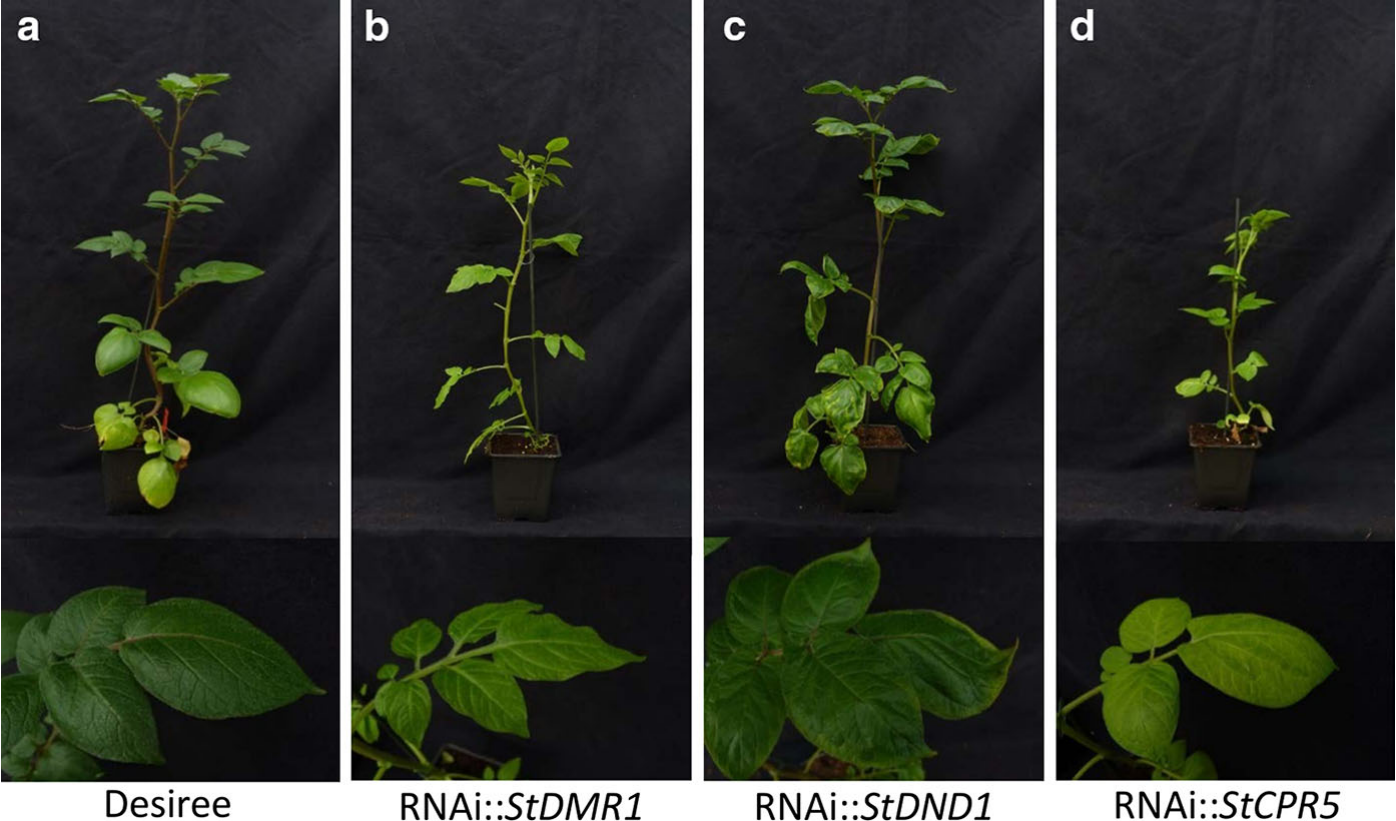
Plants and leaflets of three potato RNAi transformants compared with cv Desiree. **a** Potato cv. Desiree. **b** *StDMR1*-silenced transformant, showing a dwarf phenotype and *light green* leaves. **c** *StDND1*-silenced transformant showing auto-necrotic spots on older leaves. **d** *StCPR5*-silenced transformant showing dwarfing and *light green* leaves

Altered phenotypic characteristics in non-inoculated plants were observed for some RNAi transformants, such as reduced growth (dwarfing), necrosis, and lighter green leaves (colour loss, Table 2). *StDMR1*-silenced plants showed reduced growth and light green leaves compared with cv. Desiree (Fig. 3a, b), whereas *StDND1*-silenced plants displayed auto-necrotic spots only in the leaves of older plants (Fig. 3c). Plants of a few well-silenced *StDND1*-tranformants also showed dwarfing, but not as severe as that of *StDMR1*-silenced transformants. The light green leaf colour and dwarfing were observed for plants of all of the well-silenced RNAi::*StCPR5* transformants (Fig. 3d), and these plants were susceptible to late blight (Fig. 2a).

## Discussion

### Impaired potato *S*-genes: a new source of durable resistance against *P. infestans*?

Potato is the third largest food crop in the world, with efficient usage of resources and high nutritional quality. For over 180 years, the most devastating disease in potato is late blight disease, caused by *P. infestans*, which costs billions of Euros annually for crop protection to prevent crop losses. Many potato *R*-genes have been cloned and characterized, and some *R*-genes have been used in breeding. Still, durable resistant potato varieties have not been generated, reflecting new virulent *P. infestans* races that render resistance genes ineffective. Only a few potato varieties carrying multiple *R*-genes show a sufficient level of durable resistance, such as the cultivar Sarpo Mira, which possesses multiple major *R*-genes (Rietman et al. 2012; Tomczyńska et al. 2014). Thus, pyramiding *R*-genes show the potential to achieve durable resistance.

The results of the present study showed that silencing the potato orthologues of six *A. thalianaS*-genes resulted in complete or partial resistance to late blight in potato. These six *S*-genes were not originally identified as susceptibility factors towards late blight. Thus, these results showed that orthologues of the *S*-genes in *A. thaliana* might be functionally conserved across plant species. Furthermore, the impairment of orthologues of the same *S*-gene in different plant species could potentially result in resistance to different pathogens.

Because the *S*-gene concept is relatively new, it is not clear whether the resistance resulting from impaired *S*-genes is durable. Several natural loss-of-function alleles of plant *S*-genes have been identified in agriculture as providing durable disease resistance (Pavan et al. 2010). A well-known example is the barley *mlo* mutant for non-race specific resistance to the powdery mildew *B. graminis*, which has been successfully used in European agriculture for more than 35 years (Büschges et al. 1997; Lyngkjær et al. 2000). Natural mutant alleles of the *MLO* gene have been identified in several plant species, such as tomato (Bai et al. 2008), pea (Pavan et al. 2011) and cucumber (Berg et al. 2015), which confer resistance against different powdery mildew species. Whether impaired potato *S*-genes are a new source of durable resistance to late blight needs further study. We are currently testing the RNAi transformants with different races of *P. infestans* to verify whether resistance is broad spectrum. Moreover, an examination of the resistance mechanisms associated with impaired *S*-genes might shed light on the resistance spectrum and durability (van Schie and Takken 2014).

### Fitness costs associated with impaired potato *S*-genes

Plant *S*-genes exploited by pathogens during infection might have other intrinsic functions that are important for the plant. Therefore, impairing the function of particular *S*-genes might have adverse effects. Prior to using impaired *S*-gene alleles in crop breeding, potential negative side effects should be identified and reduced or even prevented. Mutant alleles of the 11 *S*-genes, except *DMR6* and *PMR4*, were associated with phenotypic changes in *A. thaliana* (Table 1). Remarkably, the silencing of only three of the 11 tested *S*-gene orthologues generated negative pleiotropic effects in potato (Table 2), suggesting that fitness costs could be plant species-dependent (Sun et al. 2016). For the RNAi constructs in the present study, we used the cauliflower mosaic virus 35S promoter, which is a strong and constitutively active promoter in most tissues. It is likely that the use of native *S*-gene promoters could reduce or prevent the negative side effects on plant performance. In the present study, we identified six *S*-genes whose impairment by RNAi reduced susceptibility to late blight, of which four *S*-genes were not associated with negative side effects under the conditions tested (Fig. 2; Table 2). Further studies are needed to determine whether pleiotropic effects are also absent under conditions relevant to agricultural practices.

### Strategies for exploiting impaired *S*-genes in potato breeding

As shown in the present study, silenced *S*-genes are a new source of resistance to late blight. Loss-of-function mutations in *S*-genes lead to recessively inherited resistance, which is difficult to exploit in an autotetraploid crop, such as potato. In the heterozygous (wild) potato gene pool, only dominant *R*-genes have been identified. In contrast, the resistance conferred by recessive alleles of *S*-genes is not readily selected in both the evolution and breeding of heterozygous species, such as potato. As shown in the present study, the RNAi approach can be applied to knockdown the expression of *S*-genes, which leads to resistance. RNAi has previously been applied in potato to silence the Granule-Bound Starch Synthase I (GBSSI) gene to produce potatoes with amylose-free starch (Visser et al. 1991). Currently, advanced gene-editing technologies are available for the targeted modification of all alleles of a gene. For example, TALEN-induced mutation and CRISPR-Cas9 technology have been used to generate mutant alleles of the *MLO* gene in all three genomes of allohexaploid wheat (Wang et al. 2014). In addition to these advanced techniques, F_1_ hybrid potato breeding (Lindhout et al. 2011) will be advantageous for the use of mutated *S*-genes. With a homozygous diploid line, knockout mutations can be obtained by mutagenesis, such as ethyl methanesulphonate (EMS) treatment. Thus, a realistic outlook towards late blight resistance is within reach by using *S*-gene mutants with advanced gene-editing technology or EMS in hybrid potato breeding.

## Electronic supplementary material

Below is the link to the electronic supplementary material.

Supplementary material 1 (PDF 822 kb)

## References

[CR1] Ahn IP (2007). Disturbance of the Ca^2+^/calmodulin-dependent signalling pathway is responsible for the resistance of Arabidopsis *dnd1* against *Pectobacterium carotovorum* infection. Mol Plant Pathol.

[CR2] Bai Y, Pavan S, Zheng Z, Zappel NF, Reinstädler A, Lotti C, De Giovanni C, Ricciardi L, Lindhout P, Visser R, Theres K, Panstruga R (2008). Naturally occurring broad-spectrum powdery mildew resistance in a Central American tomato accession is caused by loss of *Mlo* function. Mol Plant Microbe Interact.

[CR3] Berg JA, Appiano M, Santillán Martinez M, Hermans FWK, Vriezen WH, Visser RGF, Bai Y, Schouten HJ (2015). A transposable element insertion in the susceptibility gene *CsaMLO8* results in hypocotyl resistance to powdery mildew in cucumber. BMC Plant Biol.

[CR5] Bowling SA, Clarke JD, Liu Y, Klessig DF, Dong X (1997). The *cpr5* mutant of Arabidopsis expresses both NPR1-dependent and NPR1-independent resistance. Plant Cell.

[CR6] Brewer HC, Hawkins ND, Hammond-Kosack KE (2014). Mutations in the Arabidopsis homoserine kinase gene *DMR1* confer enhanced resistance to *Fusarium culmorum* and *F. graminearum*. BMC Plant Biol.

[CR7] Büschges R, Hollricher K, Panstruga R, Simons G, Wolter M, Frijters A, van Daelen R, van der Lee T, Diergaarde P, Groenendijk J, Töpsch S, Vos P, Salamini F, Schulze-Lefert P (1997). The barley *Mlo* gene: a novel control element of plant pathogen resistance. Cell.

[CR8] Caten CE, Jinks JL (1968). Spontaneous variability of single isolates of *Phytophthora infestans.* I. Cultural variation. Can J Bot.

[CR9] Césari S, Kanzaki H, Fujiwara T, Bernoux M, Chalvon V, Kawano Y, Shimamoto K, Dodds P, Terauchi R, Kroj T (2014). The NB-LRR proteins RGA4 and RGA5 interact functionally and physically to confer disease resistance. EMBO J.

[CR10] Chandran D, Rickert J, Cherk C, Dotson BR, Wildermuth MC (2013). Host cell ploidy underlying the fungal feeding site is a determinant of powdery mildew growth and reproduction. Mol Plant Microbe Interact.

[CR11] Chen L-Q, Hou B-H, Lalonde S, Takanaga H, Hartung ML, Qu X-Q, Guo W-J, Kim J-G, Underwood W, Chaudhuri B, Chermak D, Antony G, White FF, Somerville SC, Mudgett MB, Frommer WB (2010). Sugar transporters for intercellular exchange and nutrition of pathogens. Nature.

[CR12] Clough SJ, Fengler KA, I-c Yu, Lippok B, Smith RK, Bent AF (2000). The Arabidopsis *dnd1* “defense, no death” gene encodes a mutated cyclic nucleotide-gated ion channel. Proc Natl Acad Sci USA.

[CR14] Doehlemann G, Hemetsberger C (2013). Apoplastic immunity and its suppression by filamentous plant pathogens. New Phytol.

[CR15] Doherty CJ, Van Buskirk HA, Myers SJ, Thomashow MF (2009). Roles for *Arabidopsis* CAMTA transcription factors in cold-regulated gene expression and freezing tolerance. Plant Cell.

[CR16] Du L, Ali GS, Simons KA, Hou J, Yang T, Reddy ASN, Poovaiah BW (2009). Ca^2+^/calmodulin regulates salicylic-acid-mediated plant immunity. Nature.

[CR17] Du J, Verzaux E, Chaparro-Garcia A, Bijsterbosch G, Keizer LCP, Zhou J, Liebrand TWH, Xie C, Govers F, Robatzek S, van der Vossen EAG, Jacobsen E, Visser RGF, Kamoun S, Vleeshouwers VGAA (2015). Elicitin recognition confers enhanced resistance to *Phytophthora infestans* in potato. Nat Plants.

[CR18] Eckardt NA (2002). Plant disease susceptibility genes?. Plant Cell.

[CR19] Ellis C, Turner JG (2001). The Arabidopsis mutant *cev1* has constitutively active jasmonate and ethylene signal pathways and enhanced resistance to pathogens. Plant Cell.

[CR20] Ellis C, Karafyllidis I, Wasternack C, Turner JG (2002). The Arabidopsis mutant *cev1* links cell wall signaling to jasmonate and ethylene responses. Plant Cell.

[CR21] Flier WG, Grünwald NJ, Kroon LPNM, van den Bosch TBM, Garay-Serrano E, Lozoya-Saldaña H, Bonants PJM, Turkensteen LJ (2002). *Phytophthora ipomoeae* sp. nov., a new homothallic species causing leaf blight on *Ipomoea longipedunculata* in the Toluca Valley of central Mexico. Mycol Res.

[CR22] Fry W (2008). *Phytophthora infestans*: the plant (and *R* gene) destroyer. Mol Plant Pathol.

[CR23] Galon Y, Nave R, Boyce JM, Nachmias D, Knight MR, Fromm H (2008). Calmodulin-binding transcription activator (CAMTA) 3 mediates biotic defense responses in *Arabidopsis*. FEBS Lett.

[CR24] Genger RK, Jurkowski GI, McDowell JM, Lu H, Jung HW, Greenberg JT, Bent AF (2008). Signaling pathways that regulate the enhanced disease resistance of *Arabidopsis* “*defense*, *no death*” mutants. Mol Plant Microbe Interact.

[CR25] Govrin EM, Levine A (2000). The hypersensitive response facilitates plant infection by the necrotrophic pathogen *Botrytis cinerea*. Curr Biol.

[CR26] Hansjakob A, Riederer M, Hildebrandt U (2011). Wax matters: absence of very-long-chain aldehydes from the leaf cuticular wax of the *glossy11* mutant of maize compromises the prepenetration processes of *Blumeria graminis*. Plant Pathol.

[CR27] Haverkort AJ, Struik PC, Visser RGF, Jacobsen E (2009). Applied biotechnology to combat late blight in potato caused by *Phytophthora infestans*. Potato Res.

[CR28] Helliwell C, Waterhouse P (2003). Constructs and methods for high-throughput gene silencing in plants. Methods.

[CR30] Huibers RP, Loonen AEHM, Gao D, Van den Ackerveken G, Visser RGF, Bai Y (2013). Powdery mildew resistance in tomato by impairment of *SlPMR4* and *SlDMR1*. PLoS ONE.

[CR32] Jing H-C, Dijkwel PP (2008). *CPR5*, a Jack of all trades in plants. Plant Signal Behav.

[CR33] Jing H-C, Anderson L, Sturre MJG, Hille J, Dijkwel PP (2007). *Arabidopsis CPR5* is a senescence-regulatory gene with pleiotropic functions as predicted by the evolutionary theory of senescence. J Exp Bot.

[CR34] Jones JDG, Dangl JL (2006). The plant immune system. Nature.

[CR35] Jurkowski GI, Smith RK, I-C Yu, Ham JH, Sharma SB, Klessig DF, Fengler KA, Bent AF (2004). *Arabidopsis DND2*, a second cyclic nucleotide-gated ion channel gene for which mutation causes the “*defense*, *no death*” phenotype. Mol Plant Microbe Interact.

[CR36] Kim DS, Hwang BK (2012). The pepper *MLO* gene, *CaMLO2*, is involved in the susceptibility cell-death response and bacterial and oomycete proliferation. Plant J.

[CR37] Kim Y, Park S, Gilmour SJ, Thomashow MF (2013). Roles of CAMTA transcription factors and salicylic acid in configuring the low-temperature transcriptome and freezing tolerance of Arabidopsis. Plant J.

[CR38] Laluk K, Prasad KVSK, Savchenko T, Celesnik H, Dehesh K, Levy M, Mitchell-Olds T, Reddy ASN (2012). The calmodulin-binding transcription factor SIGNAL RESPONSIVE1 is a novel regulator of glucosinolate metabolism and herbivory tolerance in Arabidopsis. Plant Cell Physiol.

[CR39] Lindhout P, Meijer D, Schotte T, Hutten RCB, Visser RGF, van Eck HJ (2011). Towards F1 hybrid seed potato breeding. Potato Res.

[CR40] Livak KJ, Schmittgen TD (2001). Analysis of relative gene expression data using real-time quantitative PCR and the 2^−ΔΔCt^ method. Methods.

[CR41] Love AJ, Laval V, Geri C, Laird J, Tomos AD, Hooks MA, Milner JJ (2007). Components of *Arabidopsis* defense- and ethylene-signaling pathways regulate susceptibility to *Cauliflower mosaic**virus* by restricting long-distance movement. Mol Plant Microbe Interact.

[CR42] Lyngkjær M, Newton A, Atzema J, Baker S (2000). The barley *mlo*-gene: an important powdery mildew resistance source. Agronomie.

[CR43] Murashige T, Skoog F (1962). A revised medium for rapid growth and bio assays with tobacco tissue cultures. Physiol Plant.

[CR44] Nicot N, Hausman J-F, Hoffmann L, Evers D (2005). Housekeeping gene selection for real-time RT-PCR normalization in potato during biotic and abiotic stress. J Exp Bot.

[CR45] Nie H, Zhao C, Wu G, Wu Y, Chen Y, Tang D (2012). SR1, a calmodulin-binding transcription factor, modulates plant defense and ethylene-induced senescence by directly regulating *NDR1* and *EIN3*. Plant Physiol.

[CR46] Nishimura MT, Stein M, Hou B-H, Vogel JP, Edwards H, Somerville SC (2003). Loss of a callose synthase results in salicylic acid-dependent disease resistance. Science.

[CR48] Pavan S, Jacobsen E, Visser RGF, Bai Y (2010). Loss of susceptibility as a novel breeding strategy for durable and broad-spectrum resistance. Mol Breed.

[CR49] Pavan S, Schiavulli A, Appiano M, Marcotrigiano AR, Cillo F, Visser RGF, Bai Y, Lotti C, Ricciardi L (2011). Pea powdery mildew *er1* resistance is associated to loss-of-function mutations at a *MLO* homologous locus. Theor Appl Genet.

[CR50] Pel MA, Foster SJ, Park T-H, Rietman H, van Arkel G, Jones JDG, Van Eck HJ, Jacobsen E, Visser RGF, Van der Vossen EAG (2009). Mapping and cloning of late blight resistance genes from *Solanum venturii* using an interspecific candidate gene approach. Mol Plant Microbe Interact.

[CR51] Qiu Y, Xi J, Du L, Suttle JC, Poovaiah BW (2012). Coupling calcium/calmodulin-mediated signaling and herbivore-induced plant response through calmodulin-binding transcription factor AtSR1/CAMTA3. Plant Mol Biol.

[CR52] Rietman H, Bijsterbosch G, Cano LM, Lee H-R, Vossen JH, Jacobsen E, Visser RGF, Kamoun S, Vleeshouwers VGAA (2012). Qualitative and quantitative late blight resistance in the potato cultivar Sarpo Mira is determined by the perception of five distinct RXLR effectors. Mol Plant Microbe Interact.

[CR53] Streubel J, Pesce C, Hutin M, Koebnik R, Boch J, Szurek B (2013). Five phylogenetically close rice SWEET genes confer TAL effector-mediated susceptibility to *Xanthomonas oryzae* pv. *oryzae*. New Phytol.

[CR54] Su’udi M, Kim MG, Park S-R, Hwang D-J, Bae S-C, Ahn I-P (2011). *Arabidopsis* cell death in compatible and incompatible interactions with *Alternaria brassicicola*. Mol Cells.

[CR55] Sun K, Wolters AMA, Loonen AEHM, Huibers RP, van der Vlugt R, Goverse A, Jacobsen E, Visser RGF, Bai Y (2016). Down-regulation of Arabidopsis *DND1* orthologs in potato and tomato leads to broad-spectrum resistance to late blight and powdery mildew. Transgenic Res.

[CR56] Tamura K, Peterson D, Peterson N, Stecher G, Nei M, Kumar S (2011). MEGA5: molecular evolutionary genetics analysis using maximum likelihood, evolutionay distance, and maximum parsimony methods. Mol Biol Evol.

[CR57] Tomczyńska I, Stefańczyk E, Chmielarz M, Karasiewicz B, Kamiński P, Jones JDG, Lees AK, Śliwka J (2014). A locus conferring effective late blight resistance in potato cultivar Sárpo Mira maps to chromosome XI. Theor Appl Genet.

[CR58] Van Damme M, Andel A, Huibers RP, Panstruga R, Weisbeek PJ, Van den Ackerveken G (2005). Identification of *Arabidopsis* loci required for susceptibility to the downy mildew pathogen *Hyaloperonospora parasitica*. Mol Plant Microbe Interact.

[CR59] Van Damme M, Huibers RP, Elberse J, Van den Ackerveken G (2008). Arabidopsis *DMR6* encodes a putative 2OG-Fe(II) oxygenase that is defense-associated but required for susceptibility to downy mildew. Plant J.

[CR60] van Damme M, Zeilmaker T, Elberse J, Andel A, de Sain-van der Velden M, van den Ackerveken G (2009). Downy mildew resistance in *Arabidopsis* by mutation of *HOMOSERINE KINASE*. Plant Cell.

[CR61] van Schie CCN, Takken FLW (2014). Susceptibility genes 101: how to be a good host. Annu Rev Phytopathol.

[CR62] Veronese P, Nakagami H, Bluhm B, AbuQamar S, Chen X, Salmeron J, Dietrich RA, Hirt H, Mengiste T (2006). The membrane-anchored *BOTRYTIS*-*INDUCED KINASE1* plays distinct roles in Arabidopsis resistance to necrotrophic and biotrophic pathogens. Plant Cell.

[CR63] Visser RGF, Somhorst I, Kuipers GJ, Ruys NJ, Feenstra WJ, Jacobsen E (1991). Inhibition of the expression of the gene for granule-bound starch synthase in potato by antisense constructs. Mol Gen Genet.

[CR64] Vleeshouwers VGAA, van Dooijeweert W, Keizer LCP, Sijpkes L, Govers F, Colon LT (1999). A laboratory assay for *Phytophthora infestans* resistance in various *Solanum* species reflects the field situation. Eur J Plant Pathol.

[CR65] Vleeshouwers VGAA, Raffaele S, Vossen JH, Champouret N, Oliva R, Segretin ME, Rietman H, Cano LM, Lokossou A, Kessel G, Pel MA, Kamoun S (2011). Understanding and exploiting late blight resistance in the age of effectors. Annu Rev Phytopathol.

[CR66] Vogel JP, Raab TK, Schiff C, Somerville SC (2002). *PMR6*, a pectate lyase–like gene required for powdery mildew susceptibility in Arabidopsis. Plant Cell.

[CR67] Vogel JP, Raab TK, Somerville CR, Somerville SC (2004). Mutations in *PMR5* result in powdery mildew resistance and altered cell wall composition. Plant J.

[CR68] Wang Y, Cheng X, Shan Q, Zhang Y, Liu J, Gao C, Qiu J-L (2014). Simultaneous editing of three homoeoalleles in hexaploid bread wheat confers heritable resistance to powdery mildew. Nat Biotechnol.

[CR69] Waterhouse PM, Helliwell CA (2003). Exploring plant genomes by RNA-induced gene silencing. Nat Rev Genet.

[CR70] Yang T, Peng H, Whitaker BD, Conway WS (2012). Characterization of a calcium/calmodulin-regulated SR/CAMTA gene family during tomato fruit development and ripening. BMC Plant Biol.

[CR71] Yuan M, Chu Z, Li X, Xu C, Wang S (2010). The bacterial pathogen *Xanthomonas oryzae* overcomes rice defenses by regulating host copper redistribution. Plant Cell.

[CR72] Zhu S, Duwal A, Su Q, Vossen JH, Visser RGF, Jacobsen E (2013). Vector integration in triple *R* gene transformants and the clustered inheritance of resistance against potato late blight. Transgenic Res.

