# Supplementary material for: Silencing of six susceptibility genes results in potato late blight resistance
Source: Transgenic Res. 2016 May 28;25(5):731–42. doi: 10.1007/s11248-016-9964-2 (PMC5023794; doi:10.1007/s11248-016-9964-2)
Supplement: Supplementary file 1 — Supplementary material 1 (PDF 822 kb) [file 11248_2016_9964_MOESM1_ESM.pdf]

Supplementary Fig. 1. Individual phylogenetic trees of all 11 candidate susceptibility genes (*S*-genes) including homologs from tomato and potato. Sequences with an asterisk (*) which showed the highest degree of homology with the Arabidopsis *S*-gene were considered as the orthologs in potato. These are highlighted in orange, or in green in the case of multiple Arabidopsis *S*-genes in the same tree. The scale bar indicates amino acid substitutions per position. In the trees, the numbers above nodes indicate bootstrap support values based on 10,000 replicates.

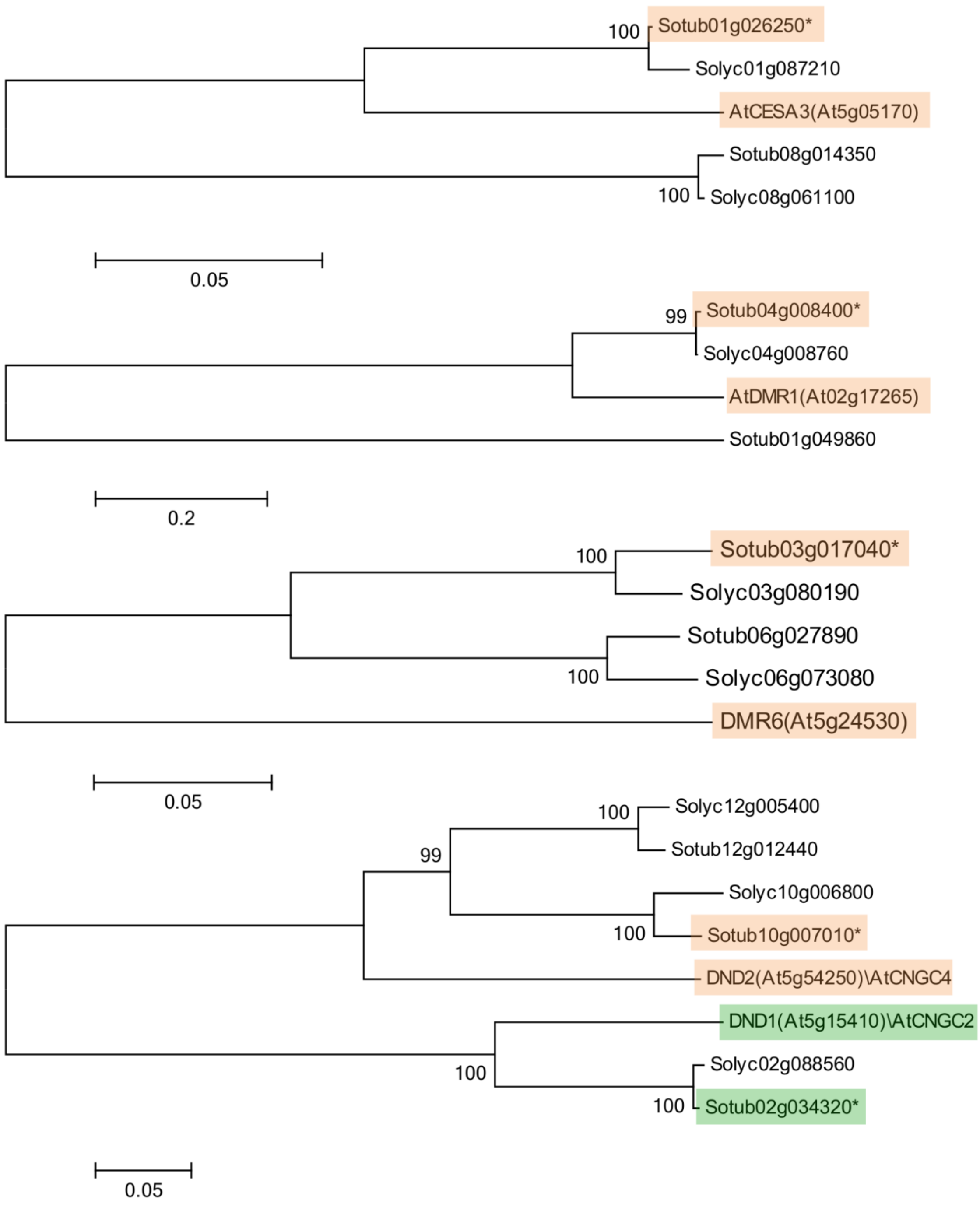

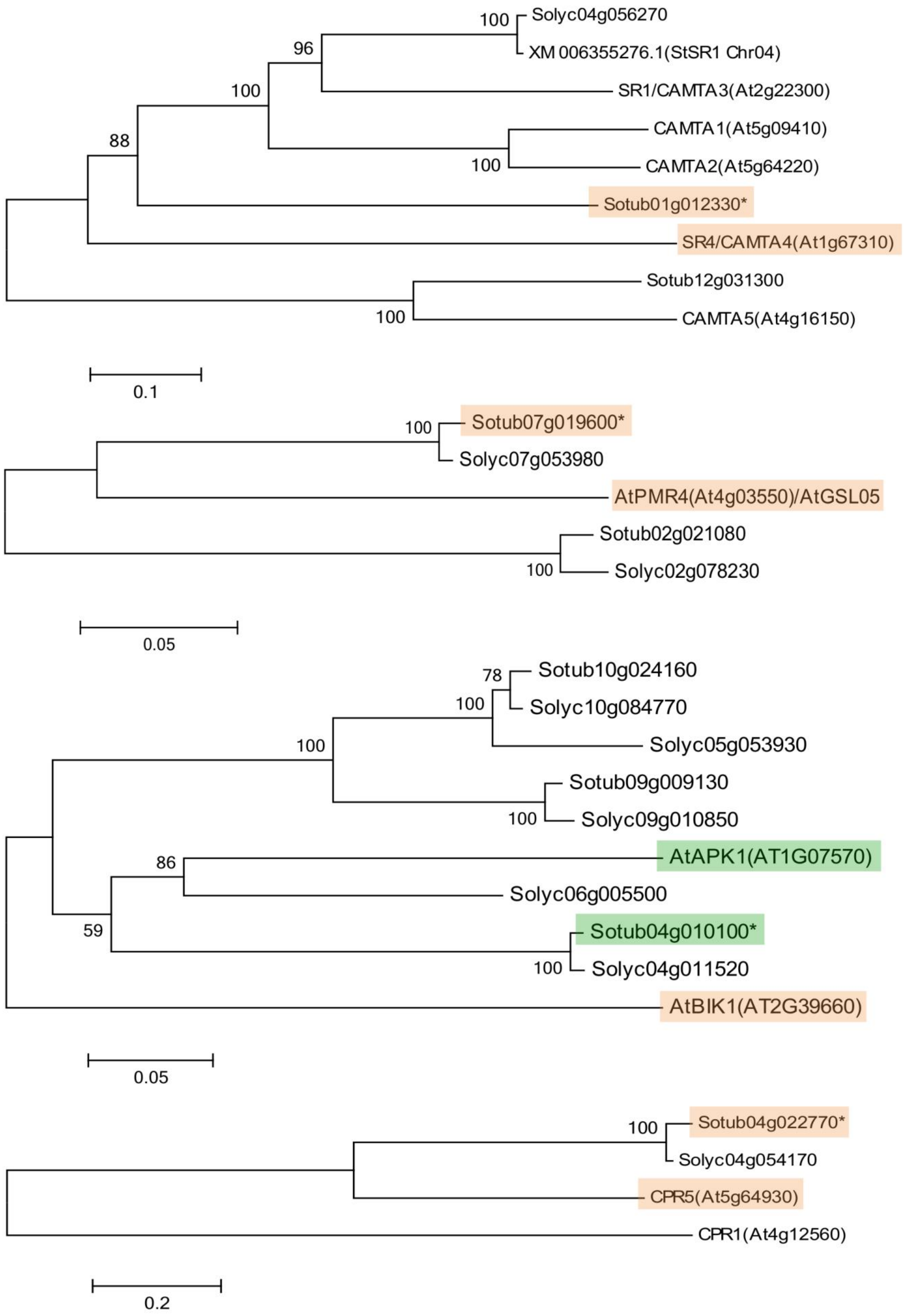

100
Solyc04g056270
96
XM 006355276.1(StSR1 Chr04)
100
SR1/CAMTA3(At2g22300)
88
CAMTA1(At5g09410)
100
CAMTA2(At5g64220)
Sotub01g012330*
SR4/CAMTA4(At1g67310)
Sotub12g031300
100
CAMTA5(At4g16150)
0.1
100
Sotub07g019600*
Solyc07g053980
AtPMR4(At4g03550)/AtGSL05
Sotub02g021080
100
Solyc02g078230
0.05
78
Sotub10g024160
100
Solyc10g084770
100
Solyc05g053930
Sotub09g009130
100
Solyc09g010850
86
AtAPK1(AT1G07570)
Solyc06g005500
59
Sotub04g010100*
100
Solyc04g011520
AtBIK1(AT2G39660)
0.05
100
Sotub04g022770*
Solyc04g054170
CPR5(At5g64930)
CPR1(At4g12560)
0.2

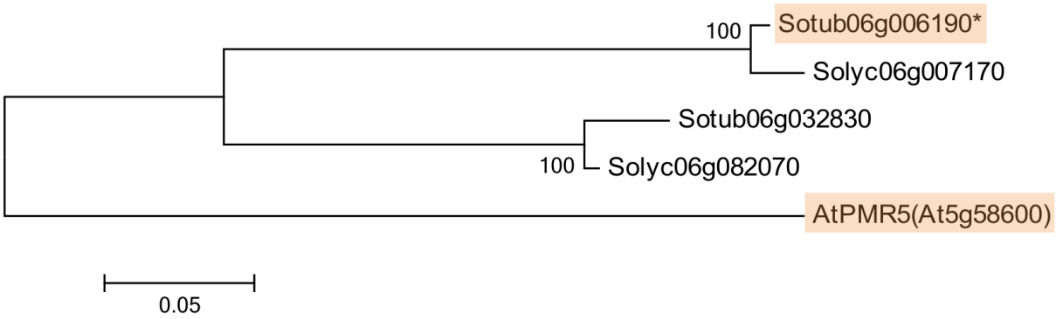
Sotub06g006190*
100
Solyc06g007170
Sotub06g032830
100
Solyc06g082070
AtPMR5(At5g58600)
0.05

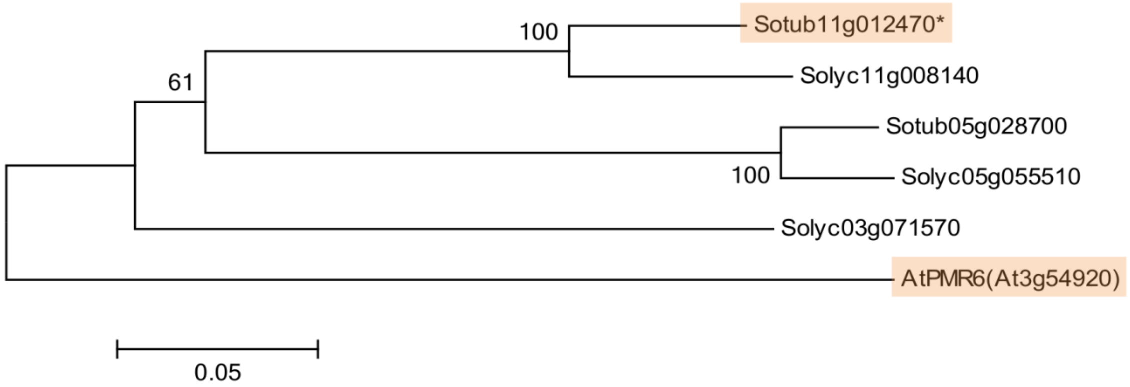
100
Sotub11g012470*
61
Solyc11g008140
Sotub05g028700
100
Solyc05g055510
Solyc03g071570
AtPMR6(At3g54920)
0.05

Supplementary Fig. 2. Information of RNAi silencing constructs and silencing effects in RNAi potato transformants. Location of the targeted regions of the RNAi constructs and qPCR fragments in 11 susceptibility genes (*S*-genes). Relative expression level of 11 potential *S*-genes in leaves of Desiree and independent potato RNAi transformants. Asterisks indicate degree of significant difference compared to Desiree plants (*p<0.05, **p<0.01, ***p<0.001)

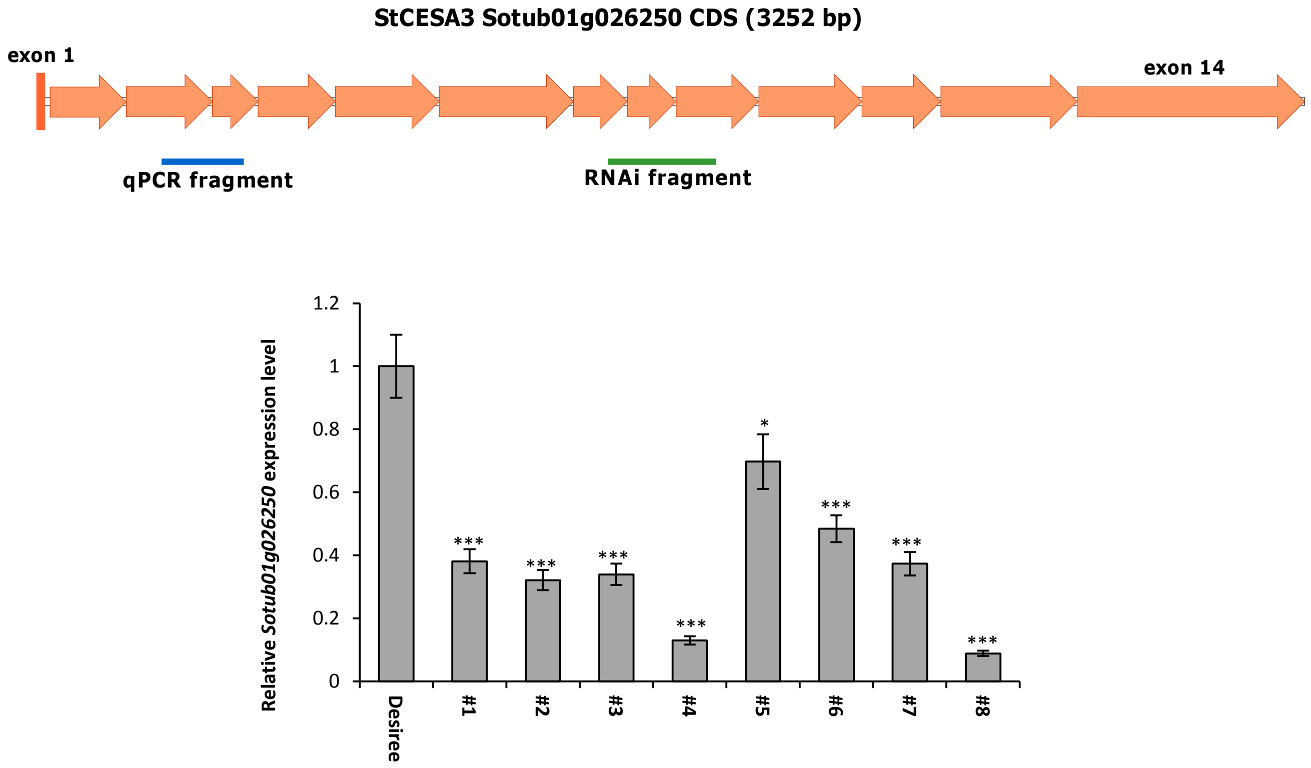

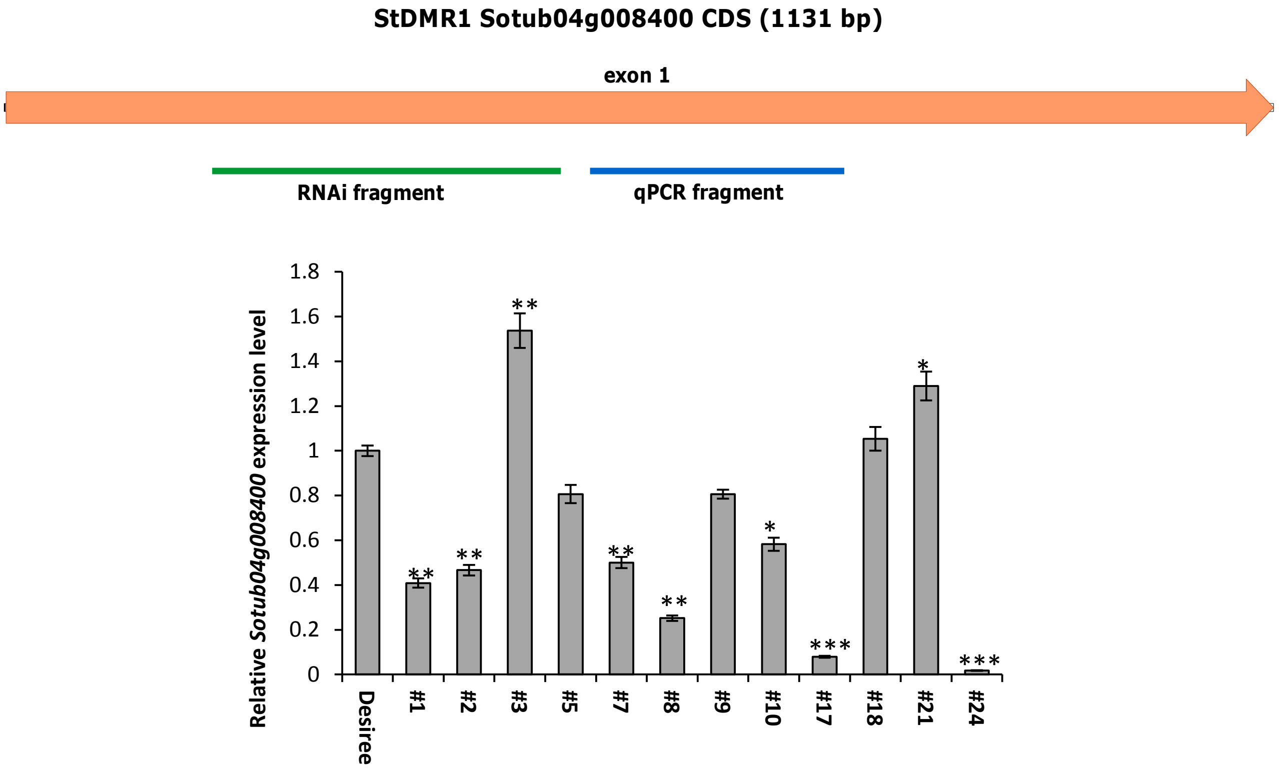
StDMR1 Sotub04g008400 CDS (1131 bp)
exon 1
RNAi fragment
qPCR fragment
Relative Sotub04g008400 expression level
0
0.2
0.4
0.6
0.8
1
1.2
1.4
1.6
1.8
Desiree
#1
#2
#3
#5
#7
#8
#9
#10
#17
#18
#21
#24
**
**
**
**
**
*
***
*
***

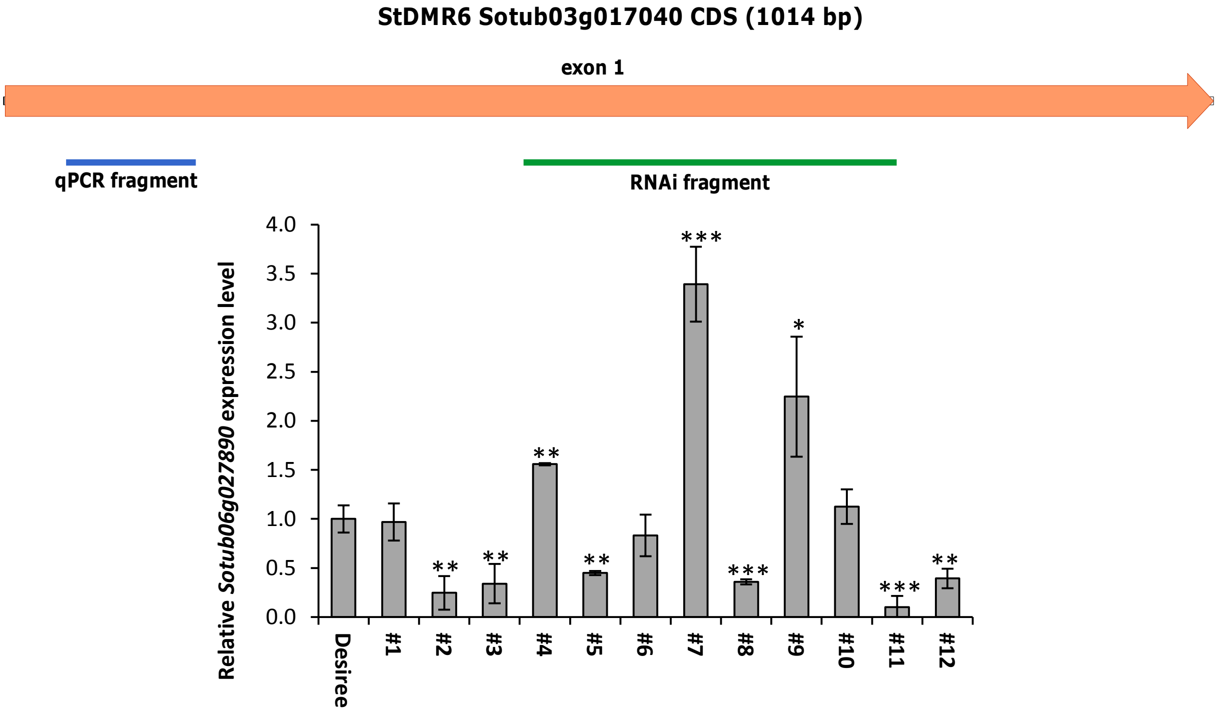
StDMR6 Sotub03g017040 CDS (1014 bp)
exon 1
qPCR fragment
RNAi fragment
Relative Sotub06g027890 expression level
0.0
0.5
1.0
1.5
2.0
2.5
3.0
3.5
4.0
Desiree
#1
#2
#3
#4
#5
#6
#7
#8
#9
#10
#11
#12
**
**
**
**
***
***
*
***
**

StDND1 Sotub02g034320 CDS (2127 bp)

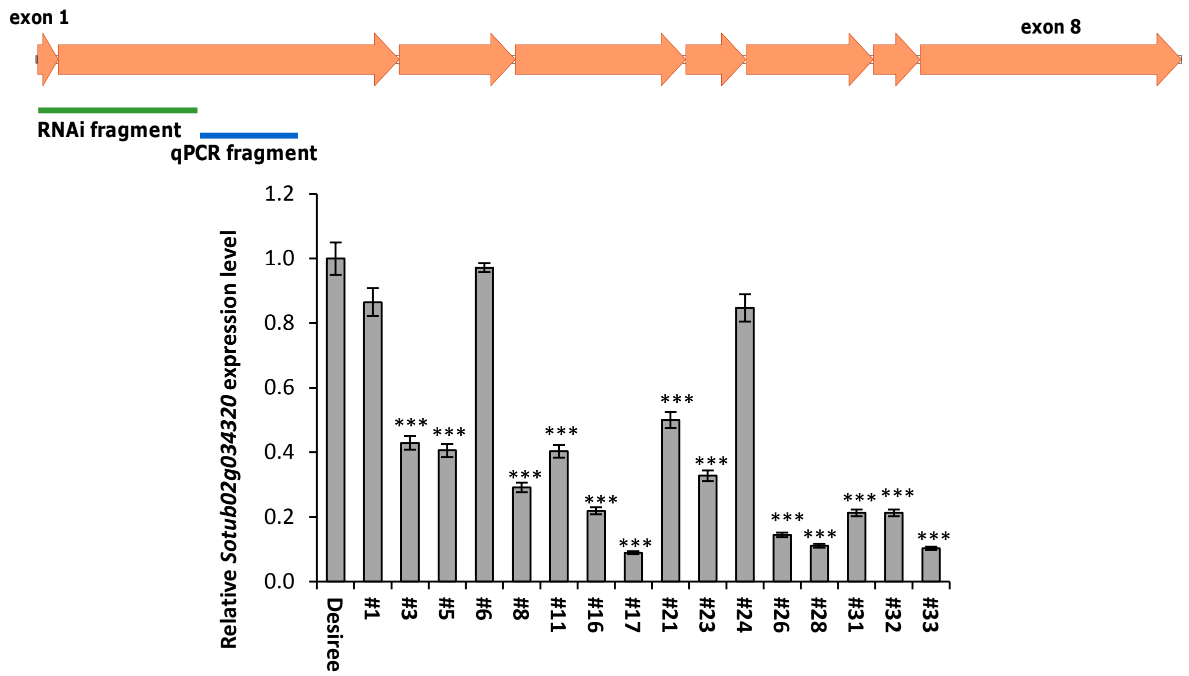

PGSC0003DMT400038758 (2903 bp)

StSR4 Sotub01g012330 exon 1

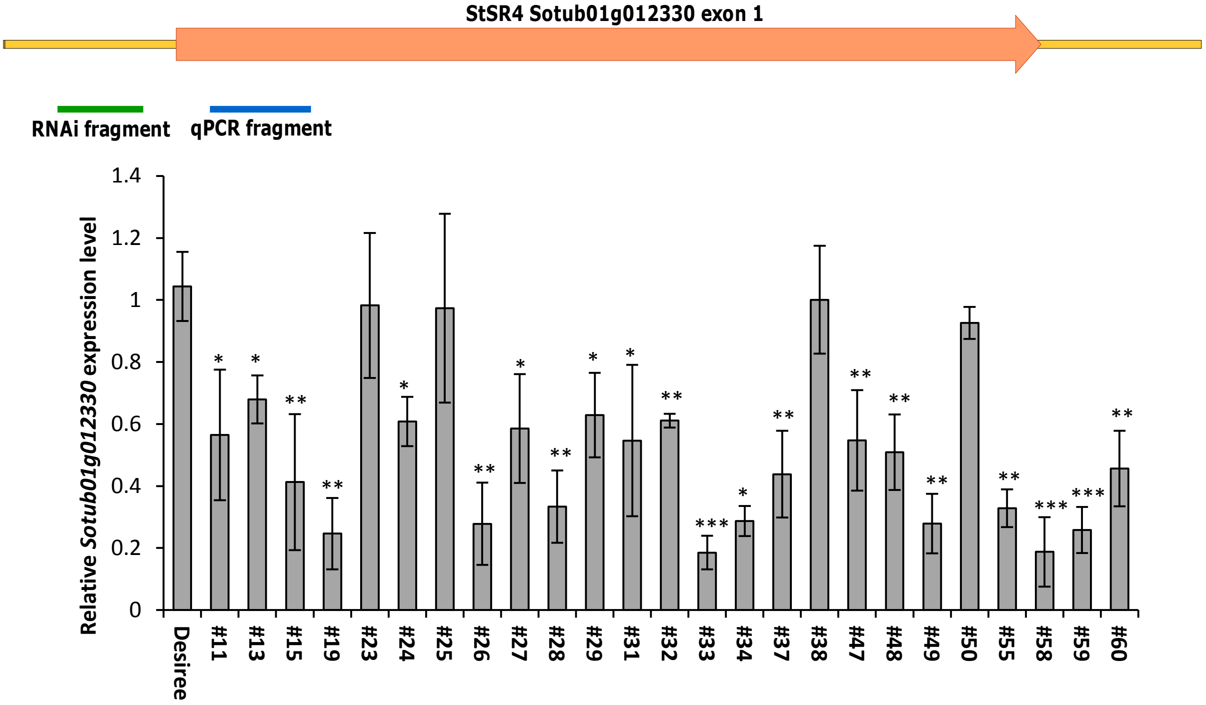

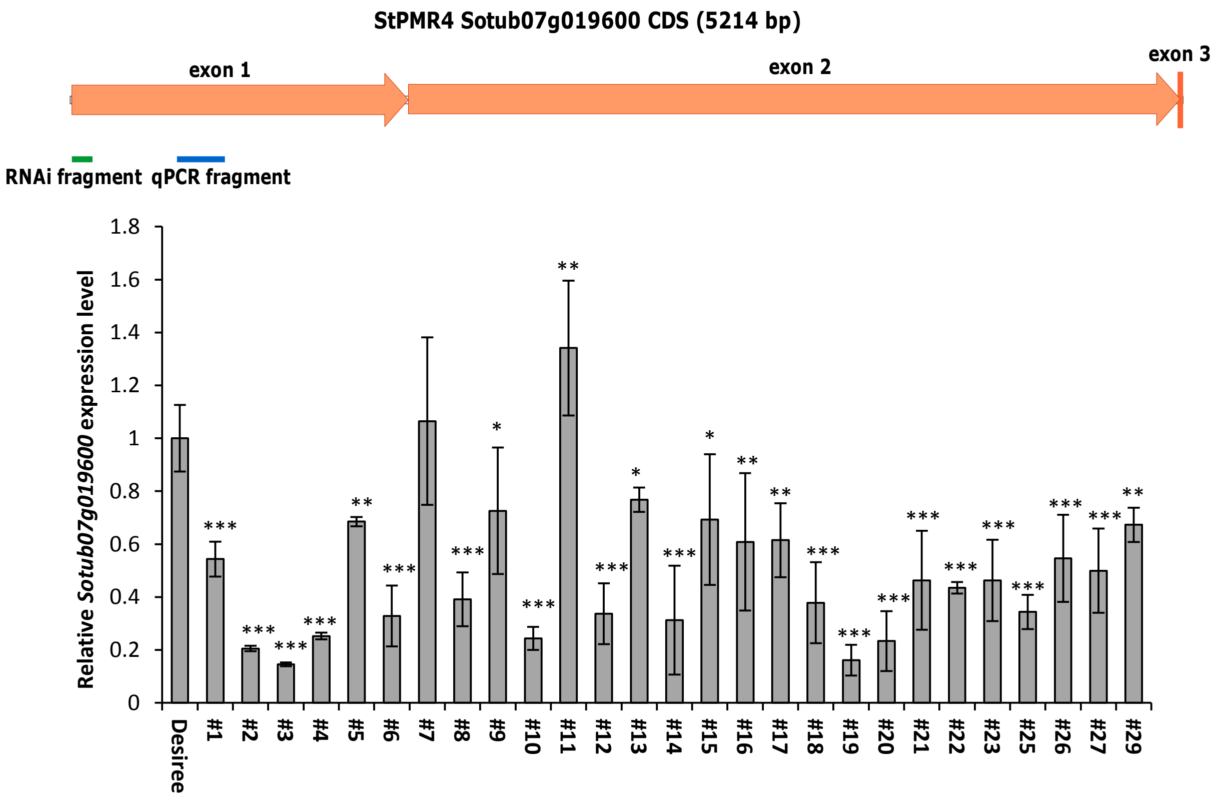
StPMR4 Sotub07g019600 CDS (5214 bp)
exon 1
exon 2
exon 3
RNAi fragment
qPCR fragment
Relative Sotub07g019600 expression level
Desiree
#1 #2 #3 #4 #5 #6 #7 #8 #9 #10 #11 #12 #13 #14 #15 #16 #17 #18 #19 #20 #21 #22 #23 #25 #26 #27 #29

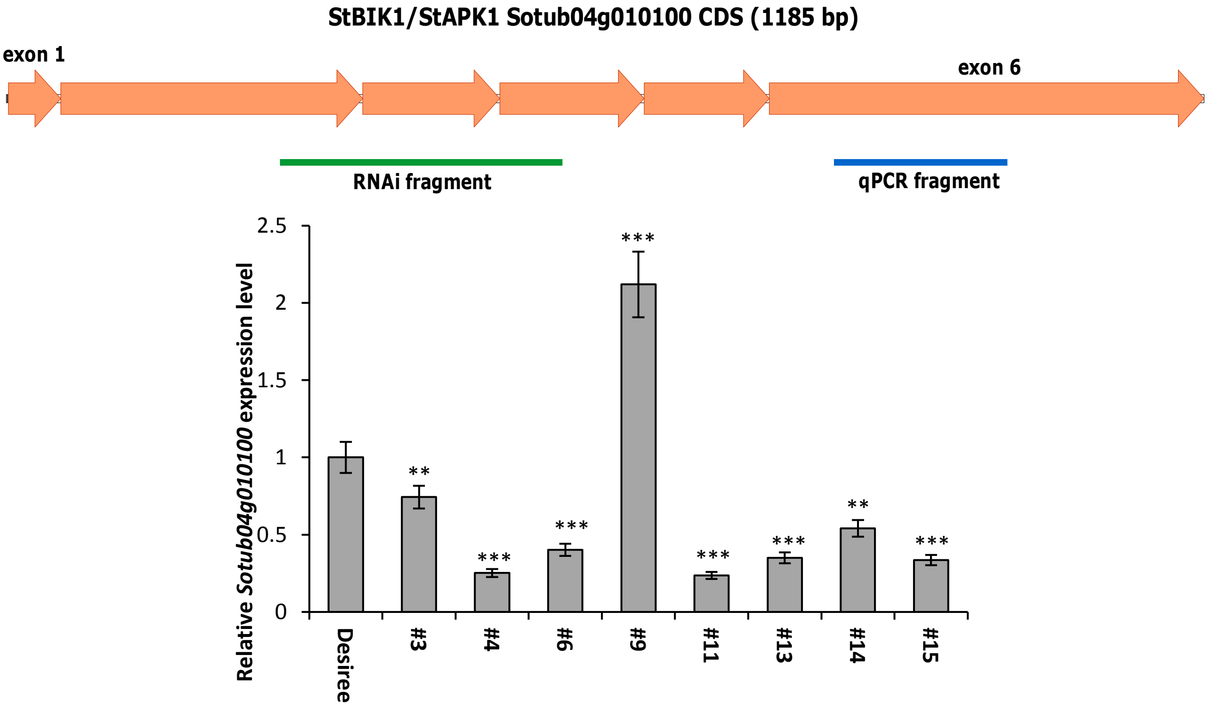
StBIK1/StAPK1 Sotub04g010100 CDS (1185 bp)
exon 1
exon 6
RNAi fragment
qPCR fragment
Relative Sotub04g010100 expression level
Desiree
#3 #4 #6 #9 #11 #13 #14 #15

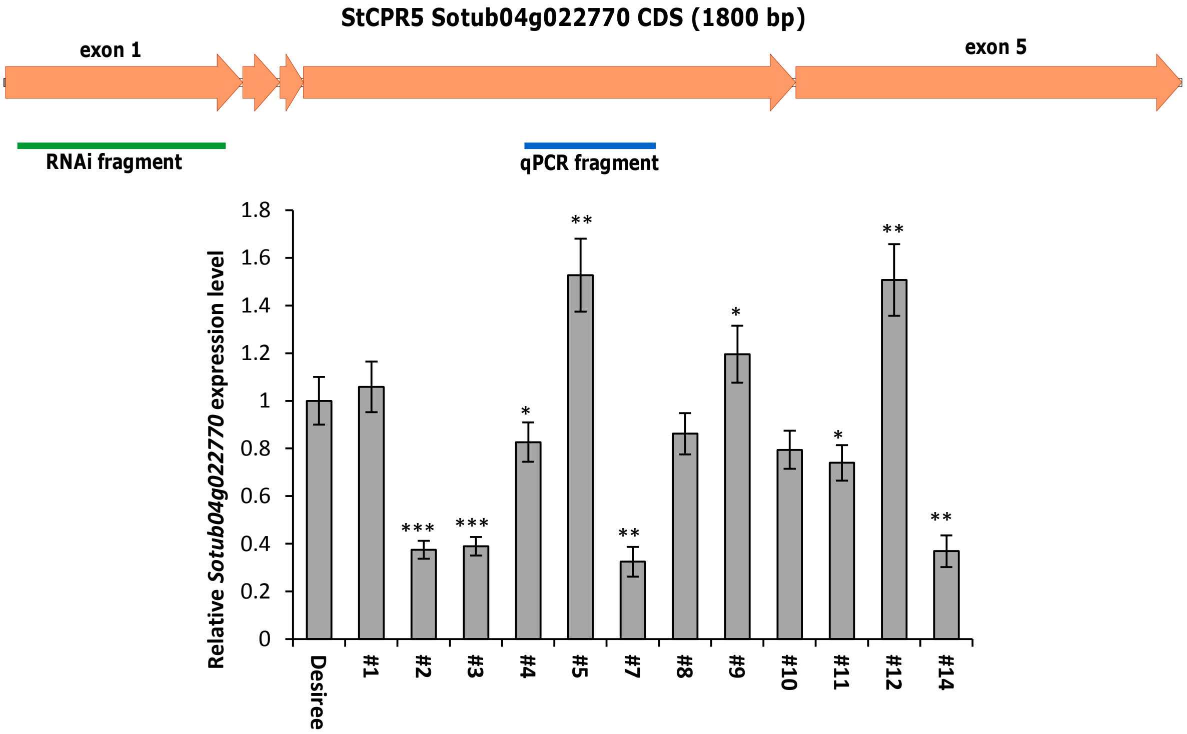

StCPR5 Sotub04g022770 CDS (1800 bp)
exon 1
exon 5
RNAi fragment
qPCR fragment
Relative Sotub04g022770 expression level
1.8
1.6
1.4
1.2
1
0.8
0.6
0.4
0.2
0
Desiree
#1
#2
#3
#4
#5
#7
#8
#9
#10
#11
#12
#14

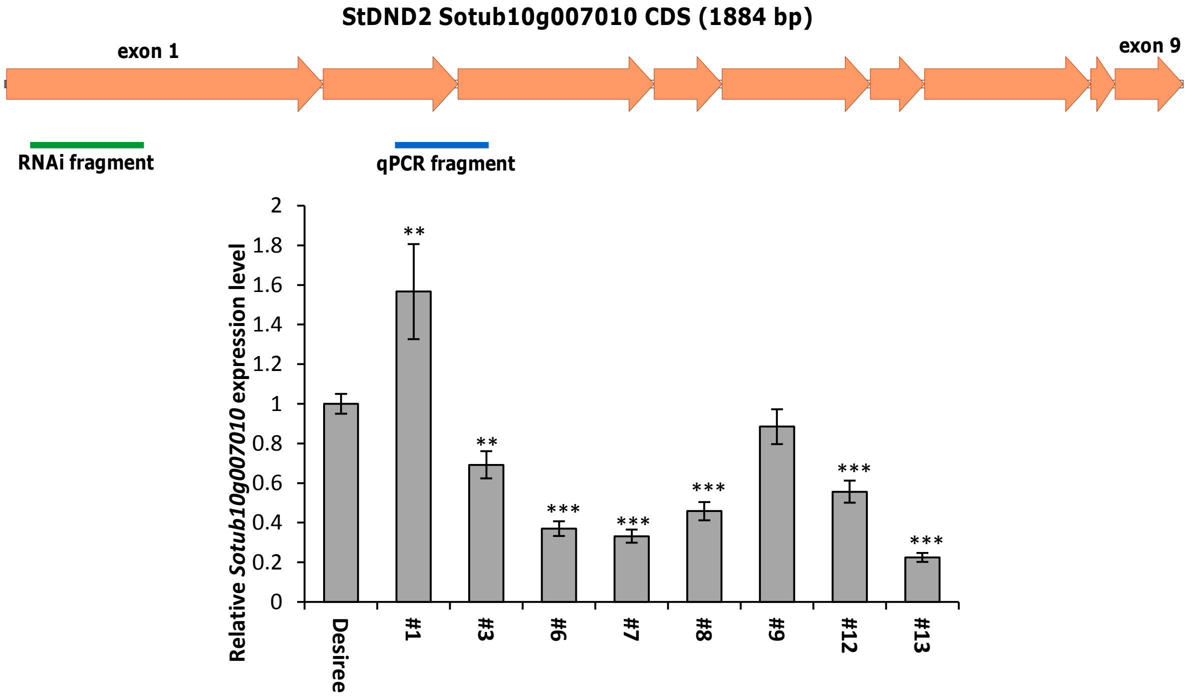

StDND2 Sotub10g007010 CDS (1884 bp)
exon 1
exon 9
RNAi fragment
qPCR fragment
Relative Sotub10g007010 expression level
2
1.8
1.6
1.4
1.2
1
0.8
0.6
0.4
0.2
0
Desiree
#1
#3
#6
#7
#8
#9
#12
#13

StPMR5 Sotub06g006190 CDS (1200 bp)

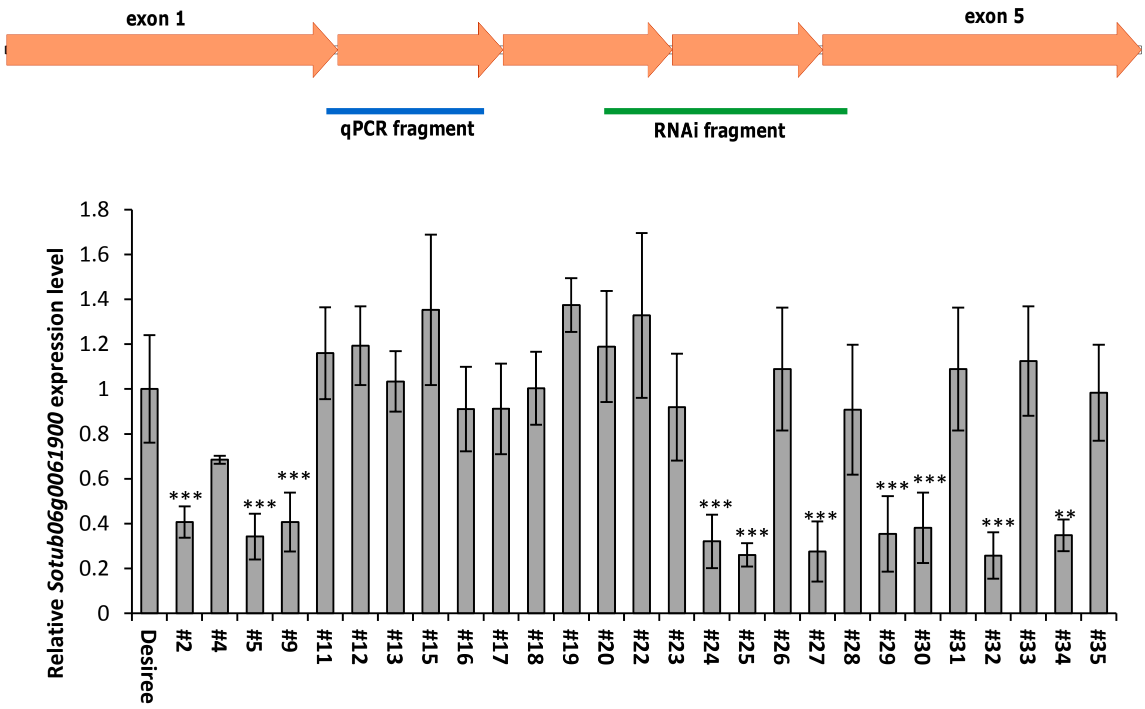

StPMR6 Sotub05g015080 CDS (1206 bp)

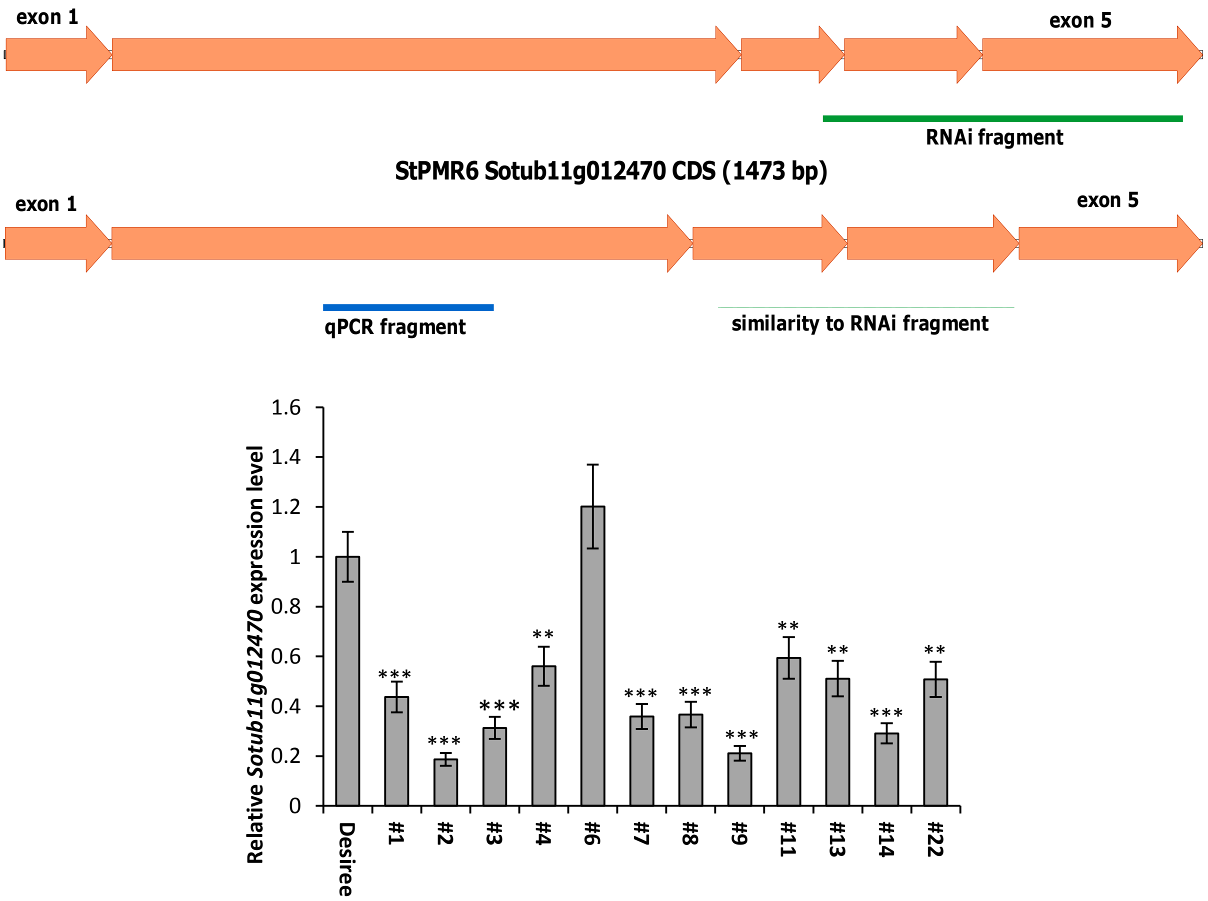

Supplementary Table 1. Primers used in this study

Primer name	Sequence (5' to 3')	Used for	Gene
Fw-SlCESA3	caccGCACACCATGGCCTGGAAATAACACC	RNAi:: SlCESA3 cloning	Solyc01g087210
Rv-SlCESA3	AAAAGCACATTGCTTCTCTCAAAGCC		
Fw-StCESA3-qPCR	GAAGCAAAAAGTGGCTGACC	Determining relative transcript levels	Sotub01g026250
Rv-StCESA3-qPCR	GATGTGTTTTGCACCACCAG		
Fw-SlDMR1	caccAGTCGTTTGCTCCGGCCACTG	RNAi:: SlDMR1 cloning	Solyc04g008760
Rv-SlDMR1	CGCCGCAGCACTAGCAGCAC		
Fw-StDMR1-qPCR	TGGACGGAAGTTGAGTGTTG	Determining relative transcript levels	Sotub01g026250
Rv-StDMR1-qPCR	CCCTCATCTTCTTGGTTGGA		
Fw-SlDND1	caccTGTCTTCTCACCAAGACGTCCGC	RNAi:: SlDND1 cloning	Solyc02g088560
Rv-SlDND1	CGATGTGTGCCGGCTGCGAC		
Fw-StDND1-qPCR	GTTCGGGCGTGTATTAGACC	Determining relative transcript levels	Sotub02g034320
Rv-StDND1-qPCR	GAATCACCGTGACGATAGCC		
Fw-SlDMR6	caccTCATCTTTCAGGGAAATCGTGAGCAG	RNAi:: SlDMR6 cloning	Solyc03g080190
Rv-SlDMR6	TGACAAAGGCGTCAGGTTGAGG		
Fw-StDMR6-qPCR	AGTTACATCCGACCCGAATC	Determining relative transcript levels	Sotub06g027890
Rv-StDMR6-qPCR	GACGGATTATTTGAGCTTGGTC		
Fw-StSR4	caccAGAATAGGTGCCTCCCGTCT	RNAi:: StSR4 cloning	PGSC0003DMT400038758/ Sotub01g012330
Rv-StSR4	GCGACCTCGAGAACTCCATA		
Fw-StSR4-qPCR	CTGGTAAAGCTCTGGCGAAC	Determining relative transcript levels	Sotub01g012330
Rv-StSR4-qPCR	GCAGTCTCCACCGATCTCTC		
	ATGAGCCTCCGGCAACGTTCAACGCCGGCGGCGAGACAAGTTTCTATAGATGAAGAACCATATAACATCATTCCGATTCATAATCTTCTAGCTGACC*	RNAi:: SlPMR4 cloning	Solyc07g053980
Fw-StPMR4-qPCR	GGGGTGAGTCAGCGAATCTA	Determining relative transcript levels	
Rv-StPMR4-qPCR	GGGCAGTACCATTCCGACTA		Sotub07g019600
Fw-SlBIK1	caccAGCTGCAAGGCCAGGGACTG	RNAi:: SlBIK1 cloning	Solyc04g011520
Rv-SlBIK1	AGCTGCCTCGAGAGCAACCTTC		
Fw-StBIK1-qPCR	TCGTCGAGTGATGGACAAAA	Determining relative transcript levels	Sotub04g010100
Rv-StBIK1-qPCR	CAGGCATTTGACAGCAAGAA		
Fw-SlCPR5	caccTCTCAACCCGTCGATCCGCC	RNAi:: SlCPR5 cloning	Solyc04g054170
Rv-SlCPR5	TGCCGAGAGGAAGTGCAAGTG		
Fw-StCPR5-qPCR	GCAATTGGCTTCTGCTTCTT	Determining relative transcript levels	Sotub04g022770
Rv-StCPR5-qPCR	CTCTCCAAAAGGTTCGCATC		
Fw-StDND2	caccTGACGATGAAGATGAAGATGATG	RNAi:: StDND2 cloning	Sotub10g007010
Rv-StDND2	CCCATTGTGCTTTAGGGTCT		
Fw-StDND2-qPCR	TTTGCCTCTTGAGAAGAATGC	Determining relative transcript levels	Sotub10g007010
Rv-StDND2-qPCR	ATTTGGCTGCCCTTTGAATA		
Fw-SlPMR5	caccTGCTTGGAGAGGTGTTGATGTCTTG	RNAi:: SlPMR5 cloning	Solyc06g007170
Rv-SlPMR5	ACGTGCCGGTGGTCCAATCG		
Fw-StPMR5-qPCR	TGAAATTCCAAGGTTCAATGG	Determining relative transcript levels	Sotub06g006190
Rv-StPMR5-qPCR	GAGGGTCACCCGTGATGTAT		

*Artificial sequence

Supplementary Table 1 (continued)

Primer name	Sequence (5' to 3')	Used for	Gene
Fw-SlPMR6	caccGGCTTGTGCAAAGGATGCCAAGATG	RNAi:: SlPMR6 cloning	Solyc05g014000
Rv-SlPMR6	TGCAACCAAGTGCACCAGCATTC		
Fw-StPMR6-qPCR	CCGTAATCCAGGAAGAACCA	Determining relative transcript levels	Sotub11g012470
Rv-StPMR6-qPCR	TATTCCCCGACGGTAAACAG		
Fw-NPT II	ACTGGGCACAACAGACAATC	Verification of transgenic plants	
Rv-NPT II	TCGTCCTGCAGTTCATTCAG		
Fw-StEF1a	ATTGGAAATGGATATGCTCCA	Normalisation in qRT-PCR assays	Sotub06g010680
Rv-StEF1a	TCCTTACCTGAACGCCTGTCA		

Supplementary Table 2. Details of susceptibility genes identified in Arabidopsis

Gene name	Subcellular location	Function	Susceptibility mechanism	References
CESA3	Golgi apparatus, endosome, integral component of membrane, plasma membrane, plasmodesma, trans-Golgi network	Cellulose synthase	Probably DAMP induced defense suppression (JA, ET)	Ellis et al. (2002); Ellis and Turner (2001)
DMR1	Chloroplast, chloroplast stroma	Homoserine kinase	Amino acid metabloism	Huibers et al. (2013); Van Damme et al. (2005); van Damme et al. (2009)
DMR6	Cytoplasm	2-oxoglutarate-Fe oxygenase	Defense suppression (SA?)	Van Damme et al. (2005); Van Damme et al. (2008)
DND1	Plasma membrane	Cyclic nucleotide-gated ion channel	Defense suppression (SA, JA), regulator NO synthesis	Ahn (2007); Clough et al. (2000); Genger et al. (2008); Govrin and Levine (2000); Jurkowski et al. (2004); Su'udi et al. (2011)
SR1	Nucleus	Ca^{2+}/calmoduline-binding transcription factor	Defense suppression (SA, ET)	Doherty et al. (2009); Du et al. (2009); Galon et al. (2008); Kim et al. (2013); Laluk et al. (2012); Nie et al. (2012); Qiu et al. (2012)
PMR4	1,3-beta-D-glucan synthase complex, Golgi apparatus, integral component of membrane, plasma membrane, plasmodesma	Callose synthase	Probably DAMP induced defense suppression (SA, JA)	Nishimura et al. (2003)
BIK1	Chloroplast, cytoplasm, nucleolus, nucleus, plasma membrane	Membrane-anchored protein kinase	Defense suppression PTI (SA?, ET)	Veronese et al. (2006)
CPR5	Chloroplast, integral component of membrane, membrane, nucleus	Transmembrane protein	Defense suppression (SA, JA, ET)	Bowling et al. (1997); Jing et al. (2007); Jing and Dijkwel (2008); Love et al. (2007)
DND2	Integral component of plasma membrane, membrane, plasma membrane	Cyclic nucleotide-gated ion channel	Defense suppression (SA, JA), regulator NO synthesis	Ahn (2007); Clough et al. (2000); Genger et al. (2008); Govrin and Levine (2000); Jurkowski et al. (2004); Su'udi et al. (2011)
PMR5	Endoplasmic reticulum, integral component of membrane	Unknown	Altered pectin composition of cell wall affecting either nutrition or endoreduplication	Chandran et al. (2013); Vogel et al. (2004)
PMR6	Anchored component of membrane, anchored component of plasma membrane, plasma membrane	Pectate lyase-like protein	Pectin accumulation in the extrahaustorial matrix possibly resulting in decreased nutrient availability to the pathogen	Chandran et al. (2013); Vogel et al. (2002)
